# Circular RNA *circPVT1* promotes nasopharyngeal carcinoma metastasis via the β-TrCP/c-Myc/SRSF1 positive feedback loop

**DOI:** 10.1186/s12943-022-01659-w

**Published:** 2022-10-05

**Authors:** Yongzhen Mo, Yumin Wang, Yian Wang, Xiangying Deng, Qijia Yan, Chunmei Fan, Shuai Zhang, Shanshan Zhang, Zhaojian Gong, Lei Shi, Qianjin Liao, Can Guo, Yong Li, Guiyuan Li, Zhaoyang Zeng, Weihong Jiang, Wei Xiong, Bo Xiang

**Affiliations:** 1 NHC Key Laboratory of Carcinogenesis and Hunan Key Laboratory of Cancer Metabolism, Hunan Cancer Hospital and Affiliated Cancer Hospital of Xiangya School of Medicine, Central South University, Changsha, China; 2 Key Laboratory of Carcinogenesis and Cancer Invasion of the Chinese Ministry of Education, Cancer Research Institute, Central South University, Changsha, 410078 Hunan China; 3 Department of Otolaryngology Head and Neck Surgery, Xiangya Hospital, Central South University, Changsha, 410078 Hunan China; 4 National Clinical Research Center for Geriatric Disorders (Xiangya Hospital), 87 Xiangya Road, Changsha, 410008 Hunan China; 5 Department of Pathology, Xiangya Hospital, Central South University, Changsha, 410078 Hunan China; 6 Department of Stomatology, Xiangya Hospital, Central South University, Changsha, Hunan China; 7 Department of Oral and Maxillofacial Surgery, The Second Xiangya Hospital, Central South University, Changsha, 410011 Hunan China; 8 Department of Medicine, Dan L Duncan Comprehensive Cancer Center, Baylor College of Medicine, Houston, TX USA

**Keywords:** Nasopharyngeal carcinoma, circPVT1, c-Myc, β-TrCP, SRSF1, Metastasis

## Abstract

**Background:**

Circular RNAs (circRNAs) act as gene expression regulators and are involved in cancer progression. However, their functions have not been sufficiently investigated in nasopharyngeal carcinoma (NPC).

**Methods:**

The expression profiles of circRNAs in NPC cells within different metastatic potential were reanalyzed. Quantitative reverse transcription PCR and in situ hybridization were used to detect the expression level of *circPVT1* in NPC cells and tissue samples. The association of expression level of *circPVT1* with clinical properties of NPC patients was evaluated. Then, the effects of *circPVT1* expression on NPC metastasis were investigated by in vitro and in vivo functional experiments. RNA immunoprecipitation, pull-down assay and western blotting were performed to confirm the interaction between *circPVT1* and β-TrCP in NPC cells. Co-immunoprecipitation and western blotting were performed to confirm the interaction between β-TrCP and c-Myc in NPC cells.

**Results:**

We find that *circPVT1*, a circular RNA, is significantly upregulated in NPC cells and tissue specimens. In vitro and in vivo experiments showed that *circPVT1* promotes the invasion and metastasis of NPC cells. Mechanistically, *circPVT1* inhibits proteasomal degradation of c-Myc by binding to β-TrCP, an E3 ubiquiting ligase. Stablization of c-Myc by *circPVT1* alters the cytoskeleton remodeling and cell adhesion in NPC, which ultimately promotes the invasion and metastasis of NPC cells. Furthermore, c-Myc transcriptionally upregulates the expression of SRSF1, an RNA splicing factor, and recruits SRSF1 to enhance the biosynthesis of *circPVT1* through coupling transcription with splicing, which forms a positive feedback for *circPVT1* production.

**Conclusions:**

Our results revealed the important role of *circPVT1* in the progression of NPC through the β-TrCP/c-Myc/SRSF1 positive feedback loop, and *circPVT1* may serve as a prognostic biomarker or therapeutic target in patients with NPC.

**Supplementary Information:**

The online version contains supplementary material available at 10.1186/s12943-022-01659-w.

## Background

Nasopharyngeal carcinoma (NPC) is a malignant tumor that occurs in the nasopharynx and has a characterized geographical distribution, within highest incidence in South China and Southeast Asia [1]. As early symptoms of NPC are not obvious or specific, most patients have neck lymph node metastasis when they are diagnosed. Although radiotherapy combined with chemotherapy are effective for the treatment of early-stage NPC, lower rate of early diagnosis and prone to metastasis blunted the treatment effect [2–5]. Recurrence and metastasis are major reasons for treatment failure and patient death in NPC [6–9]. Deeper understanding of the molecular mechanism of NPC metastasis, and clearly identifying early diagnostic biomarkers and novel therapeutic targets have become urgent scientific challenge in the field.

Circular RNAs (circRNAs), a new class of non-coding RNAs, have been reported playing important roles in cancer progress [10–19]. However, their functions and potential mechanisms in the carcinogenesis and metastasis of NPC are still not fully understood. To investigate the role of circRNAs in NPC development, the expression profile of circRNAs in NPC cells was reanalyzed and a circRNA derived from PVT1 gene, named *circPVT1* (circBase ID: hsa_circ_0001821), was selected for further study because of its high abundance. Our in vitro and in vivo experiments showed that *circPVT1* could promote the invasion and metastasis of NPC. Further experiments demonstrated that *circPVT1* inhibited ubiquitin-mediated degradation of c-Myc by binding to β-TrCP, an E3 ubiquitin ligase for c-Myc. Stablized c-Myc then modulated the expression of genes related to cell adhesion and cytoskeleton remodeling, and promoted the migration and invasion of NPC cells. In addition, the transcription factor c-Myc could upregulate and recruit SRSF1, a splicing factor, to synergistically enhance *circPVT1* biosynthesis in a transcription/splicing coupling manner. Our data suggest that *circPVT1* is a pivotal regulator for the metastatic process of NPC, and may function as a novel biomarker/target for the diagnosis/treatment of NPC.

## Methods

### Cell lines

NPC cells 5-8F and CNE2 used in this study were cultured in RPMI-1640 medium (Gibco) containing 10% fetal bovine serum (Gibco) at 37 °C and 5% CO_2_ in a constant temperature incubator.

### Clinical NPC samples

One cohort of 60 NPC and 26 non-tumor nasopharyngeal epithelial (NPE) tissues was used for RNA extraction and quantitative real-time PCR (qRT-PCR) assay (Table S1). Another cohort of paraffin embedded sections, including 159 NPC and 29 NPE samples, was used for in situ hybridization (ISH) (Table S2). These clinical NPC samples were collected from the Affiliated Cancer Hospital of Xiangya School of Medicine, Central South University and confirmed by histopathological examination. The study was approved by the Ethics Committee of Central South University, and each participant provided informed consent.

### In situ hybridization, fluorescence in situ hybridization, and immunohistochemistry

Digoxigenin- or FITC-labeled probes (BOSTER, Wuhan, China) were used for in situ hybridization (ISH) or fluorescence in situ hybridization (FISH) to detect *circPVT1* expression in NPC tissues following the instructions provided by the manufacturers. Immunohistochemistry was performed using the Elivision™ plus (Mouse/Rabbit) immunohistochemistry (IHC) kit (Kit-9902, Maxim, China). Two specialized pathologists evaluated the staining sections independently. Semi-quantitative integral analysis was used to analyze the ISH and IHC. The *circPVT1* probe used in the study is listed in Table S3.

### Vectors, siRNA sequences, and cell transfection

To construct the overexpression vector for *circPVT1*, exon 2 of the PVT1 gene was amplified by PCR and cloned into the circular RNA expression vector pCirc (gift from Yong Li, Baylor College of Medicine, USA). *circPVT1* siRNA was purchased from Genepharma (Shanghai, China). The overexpression vector was transfected using Lipofectamine 3000 (Life Technologies, NY, USA) and siRNA transfection was performed using Hiperfect (Qiagen, Hilden, Germany). Primers used are listed in Table S3.

### qRT-PCR

The Vazyme (Nanjing, China, Vazyme) reverse transcription kit was used to reverse transcribe RNA to cDNA. SYBR Green (Bimake, Shanghai, China) was used for qRT-PCR analysis, and GAPDH were used as an internal reference. The primers used in this study were synthesized by Sangon Biotech (Shanghai, China). The sequences of primers are shown in Table S3.

### Would healing and transwell invasion assays

For wound healing assay, cells were grown in 6-well plates to near confluence and scratched using a 10 μl tip. Pictures were taken under the microscope at 0 h and 24 h. To analyze cell invasion ability, 20 μl of 10% Matrigel (BD Biosciences, NJ, USA) was added to Transwell chambers in serum-free medium. Transfected cells were seeded to Transwell with 20% FBS medium culture the bottom of 24-well plate. The number of cells crossing was counted from three randomly selected areas, and cell counts were tallied using the Image J software.

### Lung metastatic xenograft model

Five-week-old female BALB/c nude mice were raised in an SPF-free barrier environment at the Experimental Animal Center of Central South University. For lung metastasis experiments, nude mice were randomly divided into four groups (*n* = 9 per group). Each nude mouse was injected via the tail vein with 1 × 10^6^ NPC CNE2 cells transfected with the *circPVT1* overexpression vector, *circPVT1* siRNA, the empty vector, or scrambled siRNA. After eight weeks, nude mice were sacrificed by cervical dislocation. Lung tissue was removed, weighed, and imaged, and the number of nodules on the surface of the lung was recorded to assess tumor metastasis. Lung tissues were then subjected to gradient dehydration, sectioned, embedded in paraffin, and stained with H&E for histological examination.

### Footpad xenograft lymph node metastasis model

Five-week-old female BALB/c nude mice were raised in an SPF-free barrier environment at the Experimental Animal Center of Central South University. To establish a nude mice footpad xenograft lymph node metastasis model, CNE2 cells transfected with the empty vector and siRNA control, the *circPVT1* overexpression plasmid, or *circPVT1* siRNA, were injected into the footpads of mice (*n* = 7 per group). After 21 days later, mice were euthanized after excision of footpad tumors and inguinal lymph nodes for H&E staining and other subsequent analyses.

### RNA pull-down experiment

The biotin-labeled *circPVT1* probe was synthesized by Sangon Biotech, and the RNA pull-down assay was performed as previously described with minor modifications. Briefly, cells were harvested 48 h after transfection, then lysed and sonicated. The biotin-labeled *circPVT1* probe (Sangon Biotech) was incubated with cell lysates at 4 °C overnight, and then incubated with Streptavidin affinity magnetic beads for another 2 h at room temperature. The supernatant was analyzed by western blotting after washing. The *circPVT1* probe used is listed in Table S3.

### RNA immunoprecipitation (RIP)

RIP was conducted with a Magna RIP kit (Millipore) following the manufacturer’s instructions. Cells were harvested and lysed in the complete RIP lysis buffer and incubated with magnetic beads conjugated with specific antibodies or negative control IgG antibody on a rotator overnight at 4 °C. Immunoprecipitated RNA was purified and enriched to detect the target RNA by qRT-PCR.

### Liquid chromatography-mass spectrometry (LC–MS/MS)

Mass spectrometry assays were performed according to the manufacturer’s protocol with minor modifications. Briefly, CNE2 cells were transfected with the *circPVT1* overexpression plasmid or the empty vector for 48 h. Total proteins were extracted and digested with protease. Peptides were dissolved in 0.1% formic acid (solvent A) and separated by ultra-high performance liquid chromatography system Easy-NLC 1000. Finally, peptides were subjected to NSI source followed by tandem mass spectrometry (MS/MS) in Q Exactive™ Plus (Thermo Scientific, Bremen, Germany) coupled online to the UPLC database. The LC–MS/MS data were processed using the Proteome Discoverer 2.1 (Thermo Fisher Scientific, MA, USA). The mass error was set to 10 ppm for precursor ions and 0.02 Da for fragment ions. Peptides confidence was at high, and peptides ion score was set > 20. For proteins identification, at least one unique peptide with a minimum 6 amino acid length was required. For differentially expressed proteins, the fold change was set ≥ 1.50 or ≤ 0.60 (Student's t-test, *p* < 0.05). The Ingenuity Pathway Analysis (IPA) software was used to obtain enrichment pathways according to the above differentially expressed proteins.

### Immunoprecipitation

Antibodies were mixed with 50 μL protein A/G magnetic beads (Bimake, USA) and incubated for 2 h at room temperature with rotation. Cells were lysed using the IP lysis buffer with protease inhibitor (Roche, USA) and left on ice for 2 h. Lysates were centrifuged, and supernatants were incubated with antibody-coupled beads overnight at 4 °C. Then, the antibody-bead complexes are washed 4 times with pre-chilled washing buffer. The complexes are then resuspended for western blotting. The primary antibodies used are listed in Table S4.

### Immunofluorescence

Cells were fixed in 4% paraformaldehyde for 15 min and then blocked with 5% BSA firstly. Then cells were incubated with specific antibodies overnight at 4 °C, washed 3–5 times with pre-chilled 0.5 M PBS and incubated with the corresponding fluorescent secondary antibody for 1 h at 37 °C. DAPI was used to stain the nucleus for 10 min and cells were photographed under a confocal microscope (Ultra-View Vox, Perkin-Elmer, Waltham, MA, USA). The primary antibodies used are listed in Table S4.

### Western blotting

Total proteins were lysed using the RIPA buffer (Beyotime Biotechnology, Shanghai, China) containing a protease/phosphatase inhibitor cocktail (Roche Applied Sciences, Mannheim, Germany), separated by 10% SDS-PAGE, and transferred onto PVDF membrane (Millipore, Billerica, MA, USA). The membrane was blocked with 5% non-fat milk with TBST for 2 h at room temperature and incubated with the primary antibodies overnight at 4 °C. After washing, the membrane was incubated with HRP-labeled secondary antibodies (CUSBIO, Wuhan, China) for 2 h at room temperature. The proteins were then detected using ECL reagent (Millipore, Billerica, MA, USA). The primary antibodies used are listed in Table S4.

### Measurement of cellular biophysical properties

A JPK NanoWizard 4 BioScience AFM (JPK Instru-ments, Berlin, Germany) was used to optically align the probe to the cells. The probes used in this study were HYDRA6V-100NG (AppNano, CA, USA) with a nominal spring constant of 0.292 N/m. During the indentation process, the loading and retraction speeds of all experiments were maintained at ~ 2.5 μm/s to avoid viscosity effects. Measurements were made in PBS at room temperature, and the cells were plated on the bottom of the cell culture dish. After transfection of the *circPVT1* overexpression vector for 48 h, NPC cells were washed twice with PBS, fixed with 2% glutaraldehyde for 45 s and 4% polymethanol solution for 30 min. Then, NPC cells were washed five times with PBS and maintained in appropriate amount of PBS for subsequent AFM scanning. The indentation depth was at least 1 mm to better simulate physiologically occurring deformations. Imaging was performed using the QI mode, and images of the AFM scan were analyzed using JPK image processing software. The force and indentation curves from each measurement were analyzed using a Hertz model to obtain the the stiffness and adhesion for each cell.

### Statistical analysis

Statistical analysis was performed using the GraphPad Prism 8 software. Student’s t-tests were used to evaluate significant differences between any two groups of data, and one-way ANOVA was used to evaluate significant differences for multiple comparisons. All data are represented as mean ± standard deviation (SD). Differences were considered significant at *p* < 0.05.

## Results

### *circPVT1* is highly expressed in NPC and associated with poor prognosis

**Fig. 1:**
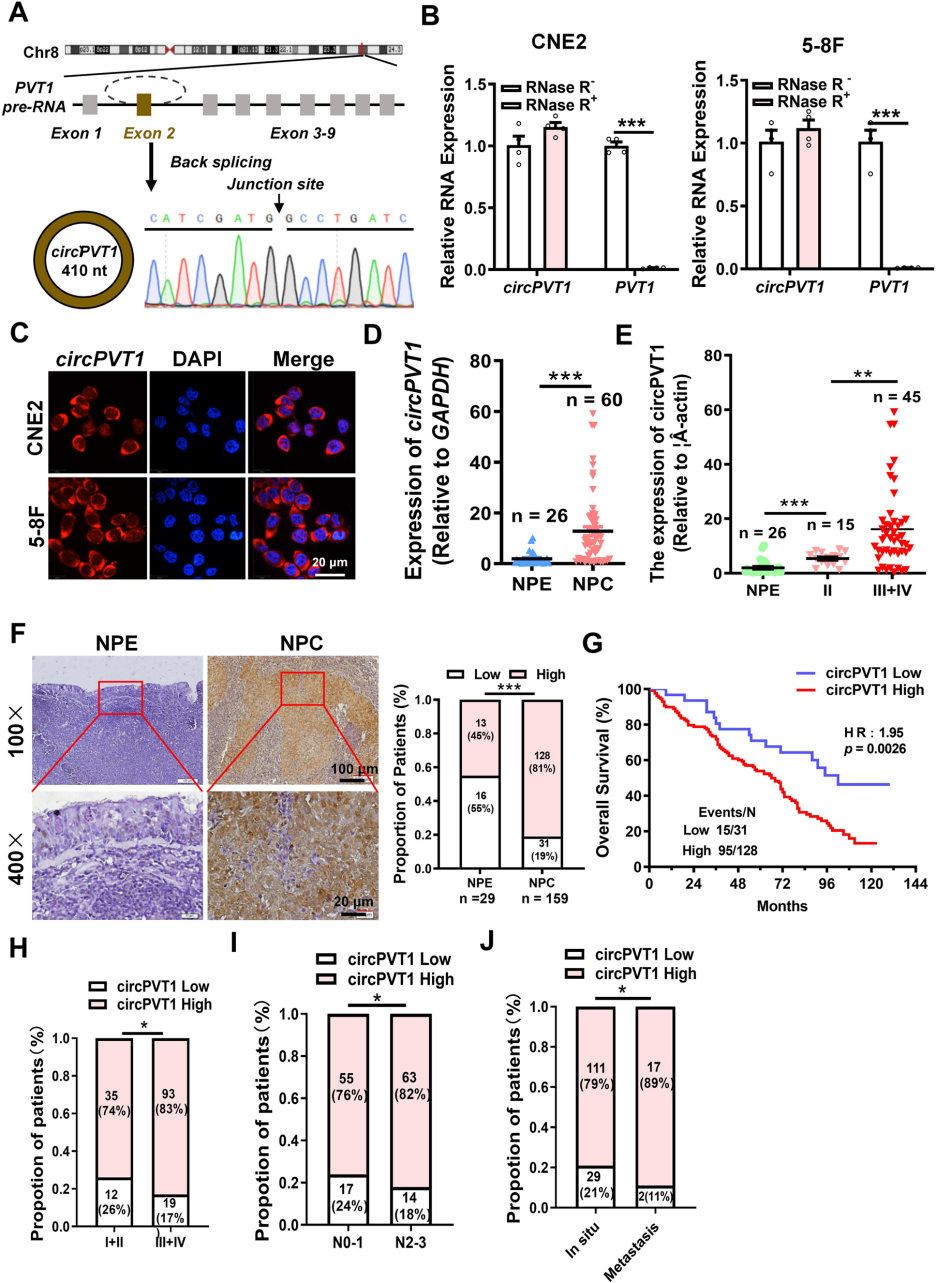
*circPVT1* is highly expressed in NPC and associated with poor prognosis. A. Schematic representation of the *circPVT1* stucture. *circPVT1* is 410 nt in length and circularly spliced from exon 2 of the PVT1 gene (RefSeq: NC_000008.11) on chromosome 8q24.21. B. *circPVT1* was more stable than the liner *PVT1* in NPC cells after RNase R treatment. ***, *p* < 0.001. C. Intracellular localization of *circPVT1* was examined using fluorescence in situ hybridization. Scale bar, 20 μm. D. Expression of *circPVT1* was examined in NPC (*n* = 60) and non-tumor nasopharyngeal epithelial tissues (NPE) (*n* = 26) by qRT-PCR. Data were represented as mean ± standard deviation (SD). ***, *p* < 0.001. E. The expression of *circPVT1* in correlation with the clinical stages in 26 NPE and 60 NPC samples. **, *p* < 0.01; ***, *p* < 0.001. F. Expression of *circPVT1* was examined in NPC (*n* = 159) and non-tumor nasopharyngeal epithelial tissues (NPE) (*n* = 29) by in situ hybridization; Scale bar: 100 × , 100 μm; 400 × , 20 μm. Left: Representative images of the *circPVT1* expression in NPC and NPE tissues. Right: The statistical results of the *circPVT1* expression in NPC and NPE tissues. ***, *p* < 0.001. G. Overall survival (OS) analysis of patients with low and high *circPVT1* staining using a Kaplan–Meier curve. H-I. The *circPVT1* staining in correlation with the clinical stages (H) and N stages (I) of patients with NPC. *, *p* < 0.05. I. High *circPVT1* expression was associated with distant metastasis in NPC patients. *, *p* < 0.05

To screen circRNAs that may regulate the progression of NPC, we reanalyzed the high-throughput RNA sequencing data (Accession numbers: GSE137543) of two NPC cell lines with different metastatic abilities (S18 cells with high metastasis potential and S26 cells with low metastasis potential). In total differentially expressed 20 circRNAs were found in the highly metastatic S18 cell line (Fold changes ≥ 1.5 and the RPM value ≥ 2), compared with S26. Combined with the other high-throughput RNA sequencing data (Accession numbers: PRJNA391554) in highly metastatic 5-8F cell line, *circPVT1* was selected for its high abundance in both sets of data (Fig. S1A-B). qRT-PCR and Sanger sequencing confirmed that *circPVT1* is backspliced by the exon 2 of PVT1, a long non-coding RNA (lncRNA) gene locate on the chromosome 8q24, and the full length of *circPVT1* is 410 nt (Fig. 1A). RNase R treatment revealed that *circPVT1* was more resistant to degradation than lncRNA *PVT1 *in NPC cells (Fig. 1B). Nucleoplasmic separation assays and RNA fluorescence in situ hybridization assays revealed that *circPVT1* was mainly localized in the cytoplasm (Fig. S1C, Fig. 1C). To examine the expression of *circPVT1* in NPC, qRT-PCR was performed in 60 NPC tissues and 26 noncancer nasopharyngeal epithelial tissues (NPE). The results showed that *circPVT1* was highly expressed in NPC samples (Fig. 1D). Moreover, the high expression of *circPVT1* was closely correlated with progression of NPC (Fig. 1E). Furthermore, the high expression of *circPVT1* in NPC tissues was further confirmed in 159 NPC and 29 noncancer NPE paraffin sections using ISH (Fig. 1F), and the high expression of *circPVT1* was positively correlated with poor prognosis (Fig. 1G), clinical stages (Fig. 1H), N stages (Fig. 1I), and distant metastasis (Fig. 1 J) in NPC patients. These data demonstrate that *circPVT1* is highly expressed in NPC and may be involved in the progression of NPC, *circPVT1* may be a potential biomarker for the detection of NPC metastasis.

### *circPVT1* promotes the invasion and metastasis of NPC cells in vitro and in vivo

**Fig. 2:**
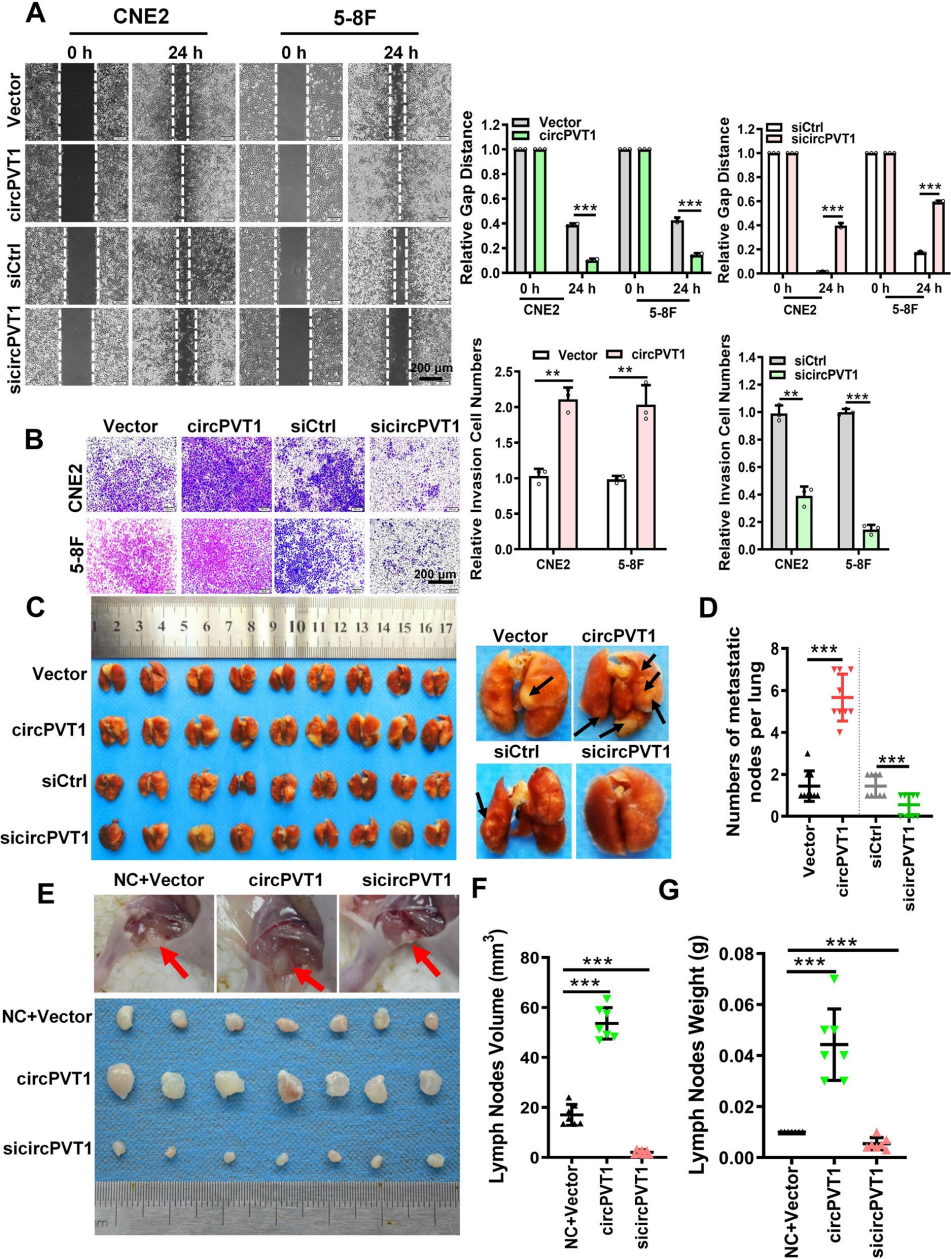
*circPVT1* promotes the migration and invasion of NPC cells in vitro and NPC metastasis in vivo. A. Wound healing assay showed that *circPVT1* promoted CNE2 and 5-8F cell migration. Images were acquired at 0 and 24 h. Data were represented as mean ± SD. ***, *p* < 0.001. B. Transwell assay showed that *circPVT1* promoted CNE2 and 5-8F cell invasion. Data were represented as mean ± SD. **, *p* < 0.05; ***, *p* < 0.001. C. Images of visible nodules on the lung surface. CNE2 cells transfected with empty vector, *circPVT1* overexpression vector, scrambled siRNA, or sicircPVT1 were injected into nude mouse tail vein (*n* = 9 for each group), and mice were sacrificed 8 weeks later. Arrows showed visible nodules on the lung surface. D. Quantification of lung metastatic nodules on lung surface. Data were represented as mean ± SD (*n* = 9 per group). ***, *p* < 0.001. E. Representative images of mice lymph nodes in the popliteal fossa of mice after injection with transfected CNE2 cells. F-G. Lymph nodes volumes (F) and lymph nodes weights (G) were measured for each group (*n* = 7 per group). Data were represented as mean ± SD. ***, *p* < 0.001

To explore the effect of *circPVT1* on the invasion and metastasis of NPC cells, *circPVT1* was specifically overexpressed or knocked down in NPC cells (Fig. S2A-B). Wound healing and Transwell assays showed that the migration and invasion ability of NPC cells was significantly enhanced after overexpression of *circPVT1* and reduced after knockdown of *circPVT1* (Fig. 2A-B). Cell Counting Kit-8 and colony formation assays showed that *circPVT1* had little effect on the growth and proliferation of NPC cells (Fig. S2C-D). We also investigated the effect of *circPVT1* on NPC cells metastasis in vivo. A lung metastatic colonization model was established through inoculating CNE2 cells transfected with the *circPVT1* overexpression vector or sicircPVT1 into nude mice tail vein (*n* = 9 per group). The number of lung nodules of the *circPVT1* overexpression group was significantly higher than that of the control group, while it was lower in the *circPVT1* knockdown group (Fig. 2C-D, Fig. S2E). NPC is prone to metastasis via lymph-vessel. To better evaluate the effect of *circPVT1* in promoting NPC cell metastasis in vivo, a nude mouse model of inguinal lymph node metastasis was constructed through footpad injection of CNE2 cells with overexpression or knockdown of *circPVT1*. The results showed that inguinal lymph nodes in the *circPVT1* group were larger than the control group (Fig. 2E-G, Fig. S2F). Immunohistochemistry showed that pan-cytokeratin-positive expression in the lymph nodes of the *circPVT1* group was significantly higher than that in the *circPVT1* knockdown group (Fig. S2G). These results show that *circPVT1* can promote the invasion and metastasis of NPC in vitro and in vivo.

### *circPVT1* promotes the migration and invasion of NPC cells by binding to β-TrCP

**Fig. 3:**
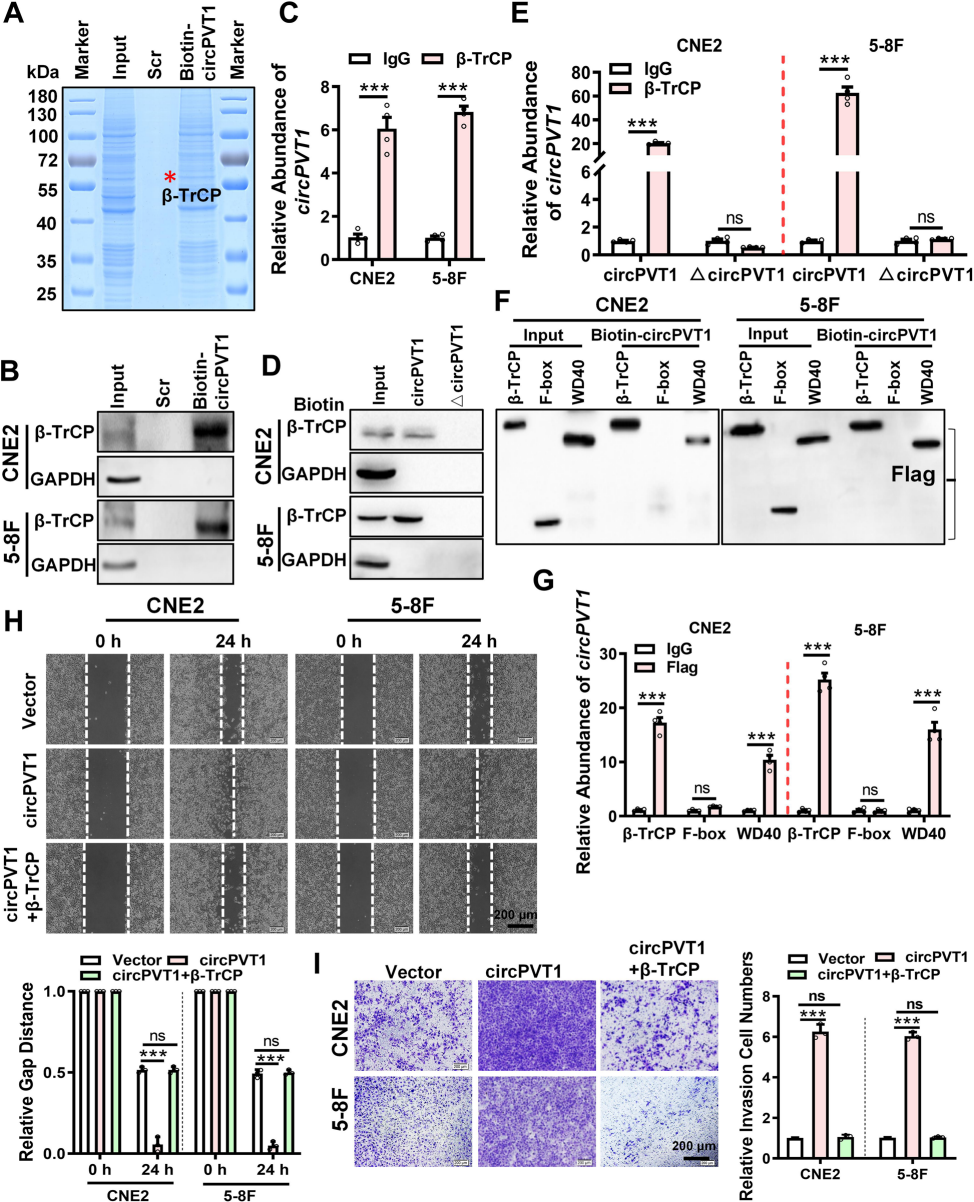
*circPVT1* promotes the migration and invasion of NPC cells by binding to β-TrCP. A. LC–MS/MS were performed to identify *circPVT1* interacting proteins in CNE2 cells after pull-down with biotin-labeled *circPVT1* probe. The unlabeled *circPVT1* probe was used as control. B. Binding of *circPVT1* and β-TrCP protein was analyzed in CNE2 and 5-8F cells after RNA pull-down with biotin-labeled *circPVT1* probe. The biotin-labeled scrambled sequences was used as a control. C. Direct binding of β-TrCP protein to *circPVT*1 was evaluated in CNE2 and 5-8F cells by RNA immunoprecipitation using anti-β-TrCP antibody, followed by qRT-PCR analysis of *circPVT1*. Data were represented as mean ± SD. ***, *p* < 0.001. D. The 230–280 nt of *circPVT1* was crucial for the interaction between *circPVT1* and β-TrCP proteins. CNE2 and 5-8F cells were transfected with the full-length *circPVT1* (*circPVT1*) or the 230–280 nt deleted mutant (△*circPVT1*). RNA pull-down assays were performed using biotin-labeled *circPVT1* probe, followed by western blotting using anti-β-TrCP antibody. E. β-TrCP protein directly binds to the 230–280 nt of *circPVT1* in CNE2 and 5-8F cells. Cells were were transfected with the full-length *circPVT1* or the deletion mutant (△*circPVT1*). RNA immunoprecipitation was performed using anti-β-TrCP antibody, followed by qRT-PCR of *circPVT1*. Data were represented as mean ± SD. ***, *p* < 0.001, ns, not significant. F. The binding between *circPVT1* and the WD40 domain of β-TrCP protein was examined in CNE2 and 5-8F cells after transfected with Flag-tagged full-length β-TrCP or truncated mutants (F-box or WD40). RNA pull-down assays were performed using biotin-labeled *circPVT1* probe, followed by western blotting using anti-Flag antibody. G. The binding between *circPVT1* and the WD40 domain of β-TrCP protein was examined in CNE2 and 5-8F cells. After transfected withFlag-tagged full-length β-TrCP or truncated mutants (F-box or WD40), RNA was immunoprecipitated using anti-Flag antibody. IgG was used as a control. Data were represented as mean ± SD. ***, *p* < 0.001, ns, not significant. H. Wound healing assay showed that overexpression of β-TrCP reverses the migrative ability of *cicPVT1* in NPC cells. Data were represented as mean ± SD. ***, *p* < 0.001, ns, not significant. I. Transwell assay showed that overexpression of β-TrCP reverses the invasive ability of *cicPVT1* in NPC cells. Data were represented as mean ± SD. ***, *p* < 0.001, ns, not significant

To elucidate the mechanism of *circPVT1* on NPC metastasis, biotin-labeled probe of *circPVT1* was used to pull-down *circPVT1* and its binding proteins. The *circPVT1* binding proteins were identified by LC–MS/MS (Fig. 3A, Table S5). Among these *circPVT1* binding proteins, according to the protein score and peptide sequence coverage, we found that an E3 ubiquitin protein ligase β-TrCP was extremely abundant (Fig. S3A). Furthermore, the interaction between *circPVT1* and β-TrCP protein was predicted, and 230–280 nt of *circPVT1* was assumed to bind to β-TrCP protein by the catRAPID website (Fig. S3B) and by the molecular docking website (http://hdock.phys.hust.edu.cn/) (Fig. S3C) according to the interaction score. RNA pull-down (Fig. 3B) and RNA immunoprecipitation (RIP) assays (Fig. 3C) confirmed the interaction between them. Then, 230–280 nt of *circPVT1* was deleted and the mutant *circPVT1* (△circPVT1) was constructed. RNA pull-down (Fig. 3D) and RNA immunoprecipitation (Fig. 3E) results verified that the mutant *circPVT1* (△circPVT1) could not bind to β-TrCP and had no effect on the migration and invasion abilities of NPC cells compared with the wild type *circPVT1* (Fig. S3D-E). These results suggest that 230–280 nt of *circPVT1* is crucial for *circPVT1* to interact with β-TrCP. Protein β-TrCP has two functional domains, the F-box domain and the WD40 repeat domain. To identify the binding region of β-TrCP that interacts with *circPVT1*, the truncated mutants, which only containing F-box domain or the WD40 repeat domain were constructed into the empty vector with Flag tag (Fig. S4A). RNA pull-down (Fig. 3F) and RNA Immunoprecipitation (RIP) (Fig. 3G) results indicated that the WD40 repeat domain but not the F-box domain of β-TrCP interact with *circPVT1*. Wound healing and transwell assays further showed that overexpression of β-TrCP could inhibit the migration and invasion of NPC cells (Fig. S4B-C) and also significantly reduced the migrative and invasive abilities of *circPVT1 *in NPC cells (Fig. S4D, Fig. 3H-I). These results suggest that *circPVT1* promotes the migration and invasion of NPC cells by binding with the WD40 repeat domain of β-TrCP through its 230–280 nt fragment.

### *circPVT1* blocks β-TrCP binding to c-Myc and inhibits the ubiquitination of c-Myc

**Fig. 4:**
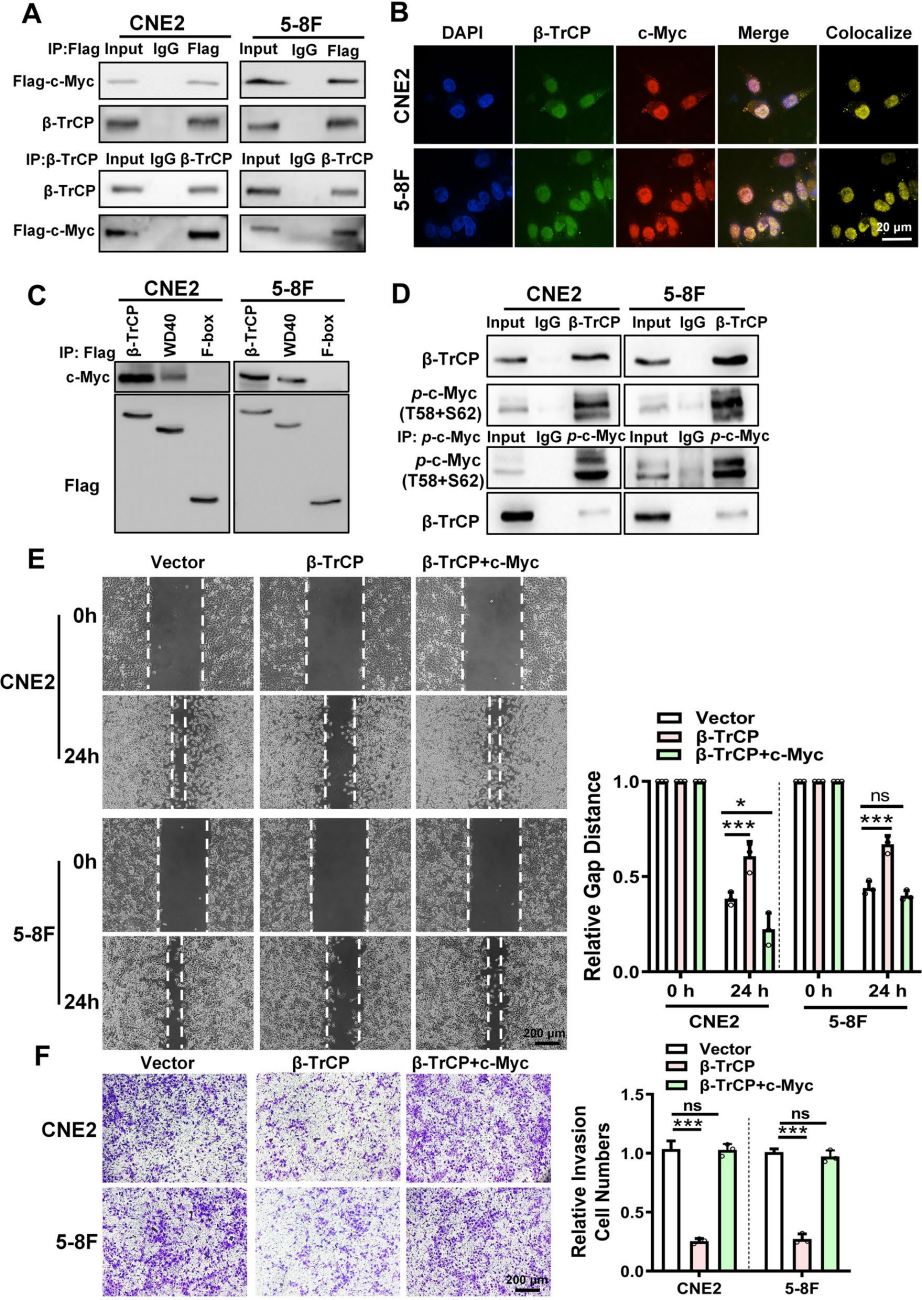
c-Myc is ubiquitinated substrate of β-TrCP. A. The interaction between β-TrCP and c-Myc proteins was examined using immunoprecipitation expriments in CNE2 and 5-8F cells after transfected with Flag tagged c-Myc. B. Immunofluorescence experiments showed that β-TrCP and c-Myc were co-localized in CNE2 and 5-8F cells. blue: DAPI-stained nucleus; green: anti-β-TrCP; red: anti-c-Myc; yellow: co-localization of β-TrCP and c-Myc. The merged image represented the overlap of DAPI, β-TrCP, and c-Myc. Scale bar, 20 μm. C. The interaction between c-Myc and the WD40 domain of β-TrCP was examined in CNE2 and 5-8F cells after transfected with Flag-tagged full-length β-TrCP or truncated fragments (F-box or WD40 domain) by immunoprecipitation using anti-Flag antibody. D. The interaction between β-TrCP and phosphorylated c-Myc proteins was examined in CNE2 and 5-8F cells by immunoprecipitation with anti-β-TrCP or anti-phosphorylated (T58 + S62) c-Myc antibodies. E. Wound healing assay showed that overexpression of c-Myc reverses the migrative ability of β-TrCP in NPC cells. Data were represented as mean ± SD. ***, *p* < 0.001, ns, not significant. F. Transwell assay showed that overexpression of c-Myc reverses the invasive ability of β-TrCP in NPC cells. Data were represented as mean ± SD. ***, *p* < 0.001, ns, not significant

To test whether *circPVT1* interferes with the E3 ubiquitin ligase function of β-TrCP to regulate its target proteins, the β-TrCP expression level was assessed in NPC cells after overexpressing or knockdown of *circPVT1*. The results showed that *circPVT1* did not affect the expression of β-TrCP **(**Fig. S5A). We hypothesized that *circPVT1* might regulate its downstream proteins expression through influencing the binding of β-TrCP to its substrates. To identify the substrates of β-TrCP in NPC cells, co-immunoprecipitation (Co-IP) was performed using β-TrCP anibody, followed by the mass spectrometry. A total of 260 proteins were identified and c-Myc exhibited high affinity towards β-TrCP (Fig. S5B, Table S6). Co-IP and immunofluorescence experiments confirmed the interaction between β-TrCP and c-Myc proteins (Fig. 4A-B). The binding domain of c-Myc and β-TrCP were predicted by Molecular Docking (Fig. S5C), which showed that c-Myc might also bind to the WD40 repeat domain of β-TrCP, which is the same domain that *circPVT1* binds to. Co-IP results confirmed the binding between the WD40 repeat domain of β-TrCP and c-Myc (Fig. 4C). c-Myc is usually phosphorylated at threonine 58 and serine 62 sites, which were recognized by E3 ubiquitin ligases. Further Co-IP experiments showed that β-TrCP prefered to interacting with the phosphorylated c-Myc protein (Fig. 4D). Overexpression of β-TrCP enhanced the ubiquitination of c-Myc and reduced the c-Myc protein level in NPC cells (Fig. S5D-E). We further co-transfected β-TrCP and c-Myc expressing vectors into the NPC cells. Wound-healing and Transwell assays showed that overexpression of β-TrCP inhibited the mobility and invasiveness of NPC cells, whereas restoration of c-Myc rescued migratory and invasive abilities of NPC cells upon overexpression of β-TrCP. These results indicated that c-Myc is the critical factor for NPC metastasis downstream of circPVT1/β-TrCP interaction.

**Fig. 5:**
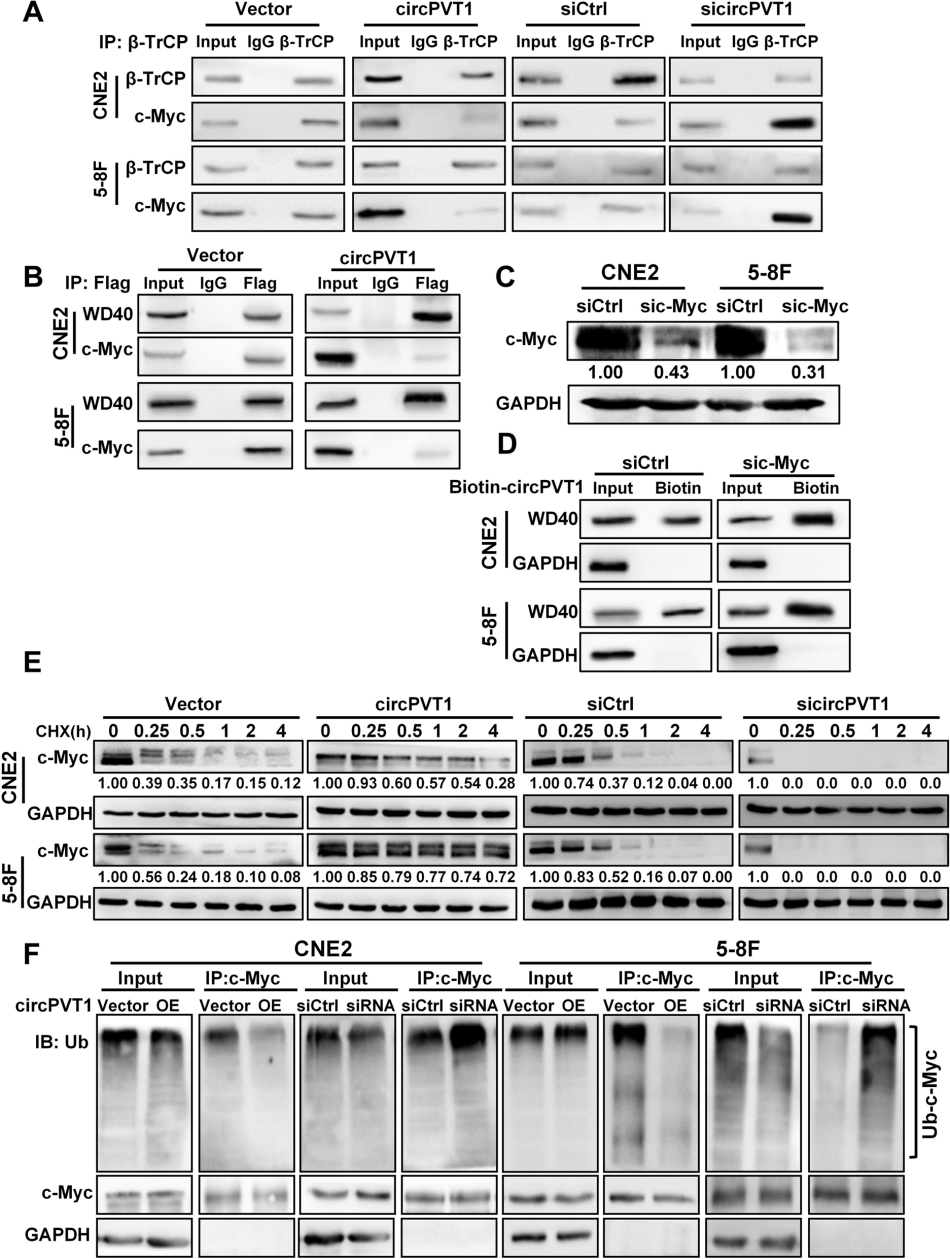
*circPVT1* blocks the binding of β-TrCP to c-Myc and inhibits the ubiquitination of c-Myc. A. The interaction between β-TrCP and c-Myc proteins was examined in CNE2 and 5-8F cells after overexpression or knockdown of *circPVT1* by immunoprecipitation using anti-β-TrCP antibody. B. The interaction between WD40 repeat domain of β-TrCP and c-Myc proteins was examined in CNE2 and 5-8F cells after overexpression of *circPVT1* by immunoprecipitation using anti-Flag antibody. C. The expression of c-Myc proteins was examined by western blotting in CNE2 and 5-8F cells after transfected with c-Myc siRNA. D. Binding of *circPVT1* and WD40 repeat domain of β-TrCP protein was detected in CNE2 and 5-8F cells after knockdown of c-Myc by RNA pull-down assays with biotin-labeled *circPVT1* probe. E. Degradation of c-Myc was detected in CNE2 and 5-8F cells after overexpression or knockdown of *circPVT1* and treatment with 50 μg/mL cycloheximide (CHX). F. The ubiquitination level of c-Myc protein was determined in CNE2 and 5-8F cells after overexpression or knockdown of *circPVT1*, immunoprecipitation with anti-c-Myc antibody, and followed by western blotting with anti-ubiquitin antibody

The above results also suggested that *circPVT1* might preoccupy the WD40 repeat domain of β-TrCP which was required for c-Myc binding in NPC cells, resulting in decreased c-Myc ubiquitination by β-TrCP. To test if *circPVT1* regulated the ubiquitination of c-Myc by β-TrCP, Co-IP was performed and the results showed that overexpression of *circPVT1* reduced the interaction between β-TrCP and c-Myc, whereas knockdown of *circPVT1* enhanced it in NPC cells (Fig. 5A). In addition, overexpression of *circPVT1* decreased the binding of c-Myc to the WD40 repeat domain of β-TrCP (Fig. 5B). Furthermore, RNA pulldown results showed that the binding of *circPVT1* to the WD40 repeat domain of β-TrCP increased after c-Myc knockdown (Fig. 5C-D). These results suggested that *circPVT1* and c-Myc competitively bound to the WD40 repeat domain of β-TrCP. When NPC cells were treated with the protein synthesis inhibitor cycloheximide (CHX) (50 μg/mL), the stability of c-Myc protein increased after overexpression of *circPVT1* and decreased after knockdown of *circPVT1* in NPC cells (Fig. 5E). Overexpression of *circPVT1* also significantly inhibited the ubiquitination of c-Myc protein, whilst knockdown of *circPVT1* had the opposite effect (Fig. 5F). The above data demonstrated that *circPVT1* stabilizd c-Myc protein in NPC cells through competitively binding with β-TrCP to block the interaction between β-TrCP and c-Myc, therefore preventing β-TrCP-mediated ubiquitination and degradation of c-Myc.

### *circPVT1* promotes the migration and invasion of NPC cells by regulating cell adhesion and cytoskeleton remodeling

**Fig. 6:**
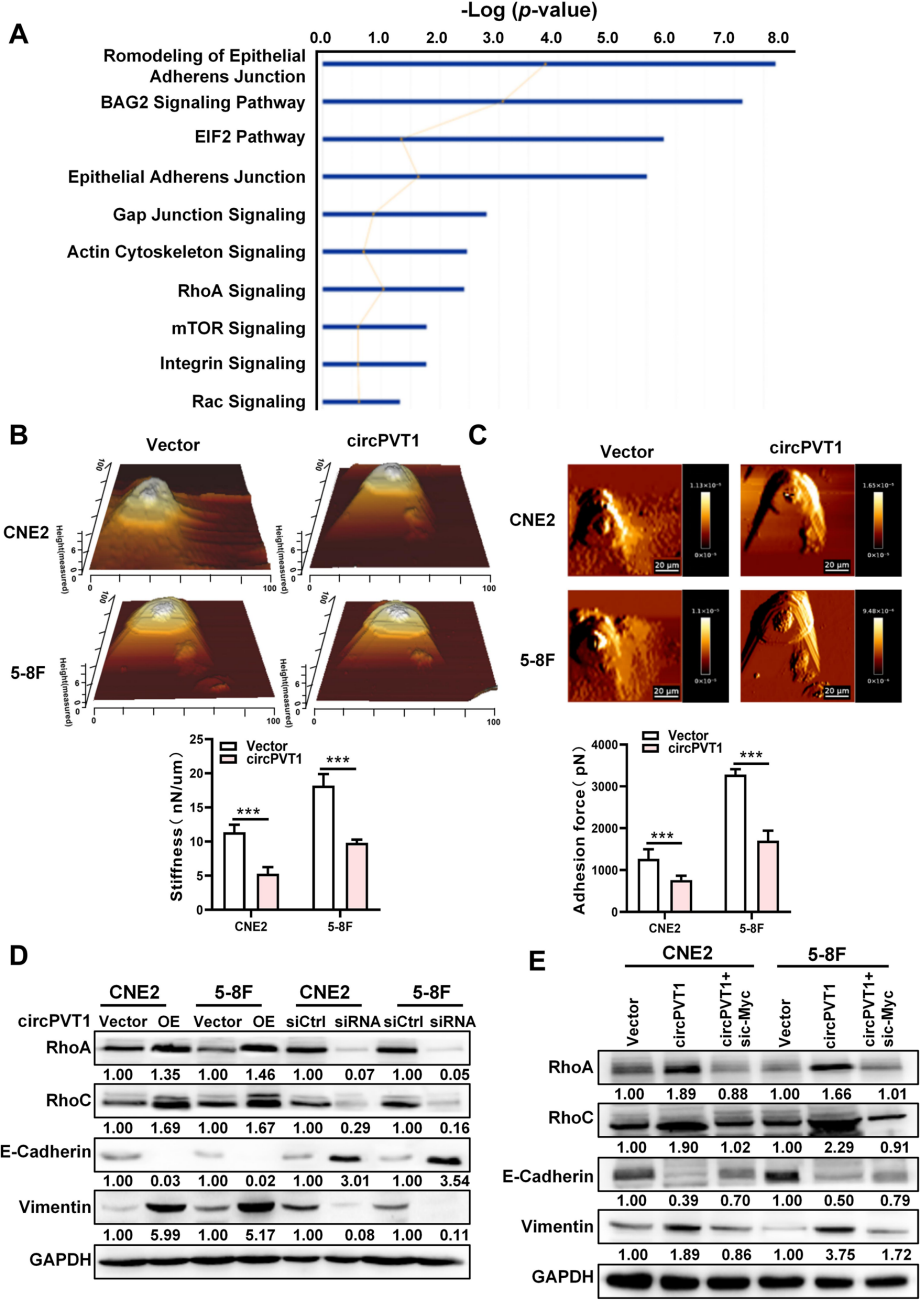
*circPVT1* promotes the migration and invasion of NPC cells by regulating cell adhesion and cytoskeleton remodeling. A. Pathways enriched by the 231 potentially regulated proteins by circPVT1 from LC–MS/MS data using the gene set enrichment analysis of the Ingenuity Pathway. B. The stiffness was measured in CNE2 and 5-8F cells after overexpresion of *circPVT1*. Top: The representative AFM deflection images; bottom: the statistical analysis of stiffness. ***, *p* < 0.001**.** C. The adhesion was measured in CNE2 and 5-8F cells after overexpresion of *circPVT1*. Top: The representative AFM deflection images; bottom: the statistical analysis of adhesion. ***, *p* < 0.001**.** D. The expression of RhoA, RhoC, E-cadherin, and vimentin proteins was examined in CNE2 and 5-8F cells after overexpression or knockdown of *circPVT1* by western blotting. E. The expression of RhoA, RhoC, E-cadherin and vimentin proteins was examined in CNE2 and 5-8F cells after co-transfected with the c-Myc siRNA and *circPVT1* overexpression vector by western blotting

To explore downstream genes which were regulated by the *circPVT1/*β-TrCP/c-Myc axis, the proteome profile of NPC cells after overexpression *circPVT1* was examined by mass spectrometry. A total of 231 significantly differentially expressed proteins were identified (Table S7). Biological functions of these proteins were analyzed by Ingenuity Pathway Analysis (IPA), the result showed that sevral signaling pathways related to cell adhesion, cell junction and cytoskeleton were enriched. (Fig. 6A). We performed upstream factor analysis of differential proteins obtained by whole-proteome mass spectrometry data, interestingly, the results showed that a large number of differential proteins were regulated by c-Myc (Fig. S6). We then assessed the stiffness and adhesion of NPC cells after overexpresion of *circPVT1* by Atomic Force Microscope (AFM). The data showed that the stiffness and adhesion of NPC cells was decreased when *circPVT1* was overexpressed in NPC cells (Fig. 6B-C). In addition, several key molecules relevant to cell adhesion junctions and cytoskeleton remodeling pathways such as RhoA, RhoC, E-cadherin, and Vimentin were also examined in NPC cells after overexpression or knockdown of *circPVT1*, the results showed that *circPVT1* could induce the expression of RhoA, RhoC, and Vimentin, whereas reduce E-cadherin expression (Fig. 6D). Furthermore, knockdown of c-Myc reversed *circPVT1* induced up-regulation of RhoA, RhoC, and Vimentin, as well as *circPVT1* reduced E-cadherin expression (Fig. 6E). These results suggest that *circPVT1* regulate cell adhesion junctions and cytoskeleton remodeling through the *circPVT1/*β-TrCP/c-Myc axis.

### c-Myc promotes *circPVT1* biosynthesis through upregulating and recruiting SRSF1

**Fig. 7:**
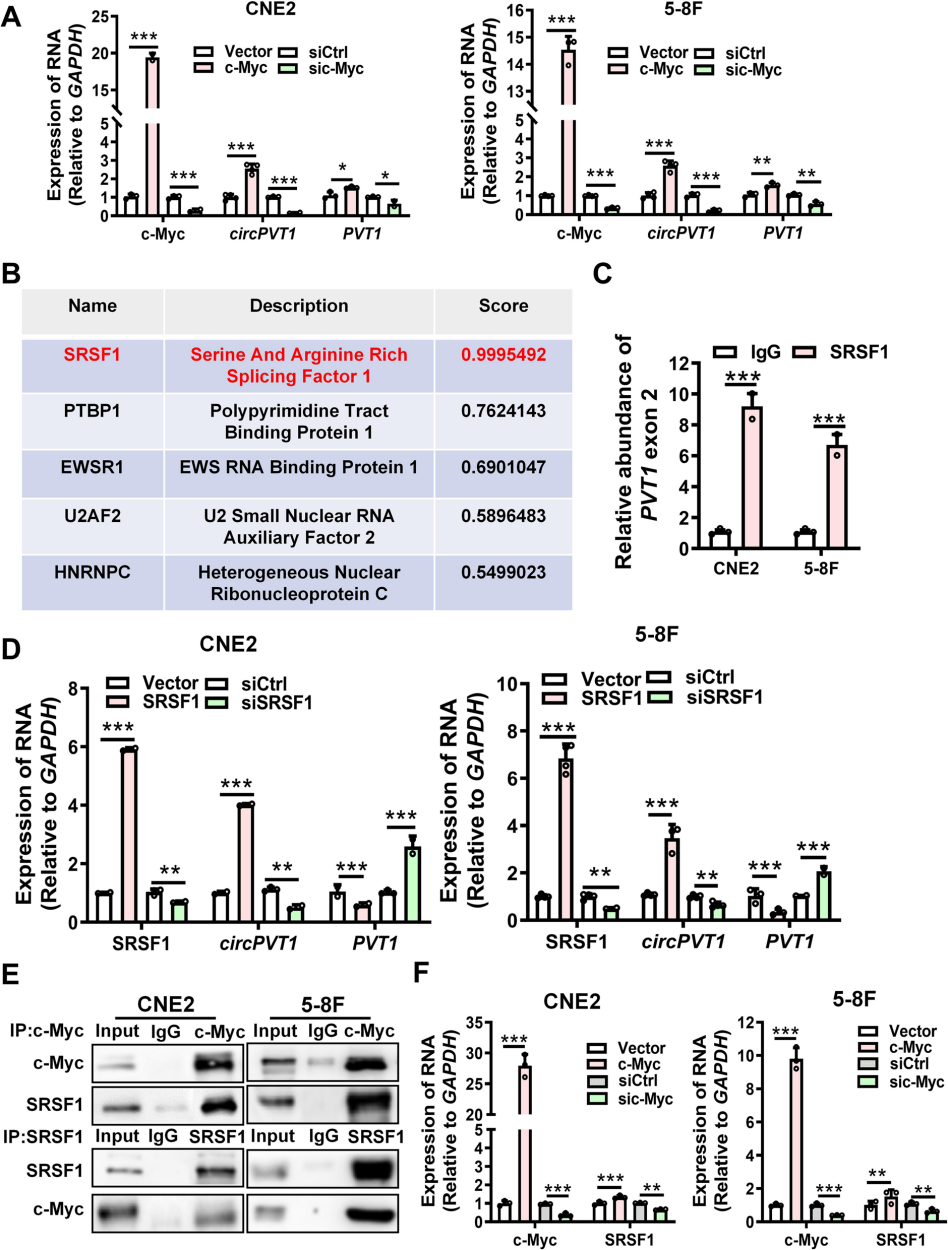
c-Myc promotes *circPVT1* generation by recruiting SRSF1 to couple transcription to splicing. A. The expression of *circPVT1* and *PVT1* in CNE2 and 5-8F cells after overexpression or knockdown of c-Myc was examined using qRT-PCR. Data were represented as mean ± SD; *, *p* < 0.05; **, *p* < 0.01; ***, *p* < 0.001. B. The top 5 splicing factors potentially binding to the exon 2 of *PVT1* were listed following analyzed online (RBPsuite: http://www.csbio.sjtu.edu.cn/bioinf/RBPsuite/). The score for SRSF1 is highest (score = 0.9995492). C. The interaction between SRSF1 protein and exon 2 of *PVT1* was evaluated in CNE2 and 5-8F cells by RNA immunoprecipitation using anti-SRSF1 antibody, followed by qRT-PCR analysis of the *PVT* exon 2*.* Data were represented as mean ± SD. ***, *p* < 0.001. D. The expresion of *circPVT1* and *PVT1* in CNE2 and 5-8F cells after overexpression or knockdown of SRSF1 was examined using RT-PCR. Data were represented as mean ± SD; **, *p* < 0.01; ***, *p* < 0.001. E. The interaction between SRSF1 and c-Myc proteins was examined in CNE2 and 5-8F cells using immunoprecipitation with anti-c-Myc antibody, followed by western blotting using anti-SRSF1 antibody. F. The expression of SRSF1 in CNE2 and 5-8F cells after overexpression or knockdown of c-Myc was examined using qRT-PCR. Data were represented as mean ± SD; **, *p* < 0.01; ***, *p* < 0.001

The expression of *circPVT1 *in NPC cells was also examined after overexpression or knockdown of c-Myc. The result of qRT-PCR showed that both *circPVT1* and linear *PVT1* were upregulated by c-Myc, indicating that c-Myc could promote the production of PVT1 precursor RNA at the transcriptional level (Fig. 7A). We analyzed the potential promoter region of PVT1 using bioinformatics tools. Three c-Myc potential binding sites were found in the PVT1 promoter region. Luciferase-reporter assay and ChIP-qPCR assays demonstrated that c-Myc binds to the PVT1 promoter region from -871 bp to -528 bp to promote the transcription of PVT1 (Fig. S7A-B). CircRNAs are product of RNA alternative splicing, and splicing factors are involved in the biogenesis of circRNAs. Bioinformatics analysis using the RBPsuite (http://www.csbio.sjtu.edu.cn/bioinf/RBPsuite/) predicted that there were several splicing factor binding sites on exon 2 of PVT1. Among these splicing factors, SRSF1 displayed the highest score (Fig. 7B). RIP experiments confirmed that SRSF1 could indeed bind to exon 2 of PVT1 (Fig. 7C). The expression of *circPVT1* was elevated following SRSF1 overexpression, while the level of *PVT1* was reduced. In contrary, knockdown of SRSF1 resulted in reduction of *circPVT1* level but elevation of *PVT1* level in NPC cells (Fig. 7D). Thus, our data suggested that SRSF1 binds to the exon 2 of *PVT1* and facilitates the biogenesis of *circPVT1* in NPC cells. Interestingly, Co-IP experiments revealed an unexpected interaction between c-Myc and SRSF1 (Fig. 7E). Moreover, c-Myc as a transcription factor could also promote the expression of SRSF1 (Fig. 7F, Fig. S7C-D). Finally, we performed RNA pull-down assay with fresh NPC samples. The results verified that *circPVT1* could bind to β-TrCP protein extracted from fresh NPC samples. Immunohistochemical results showed that the intensity of *circPVT1* was positively correlated with levels of c-Myc and SRSF1 in NPC tissues (Fig. S7E-F, Tables S8-S9). These results suggest that c-Myc not only acts as a transcription factor to promote gene transcription, but also enhances splicing of pre-RNA of *PVT1* and thus facilitates the biosynthesis of *circPVT1* by coupling transcription to splicing.

## Discussion

**Fig. 8:**
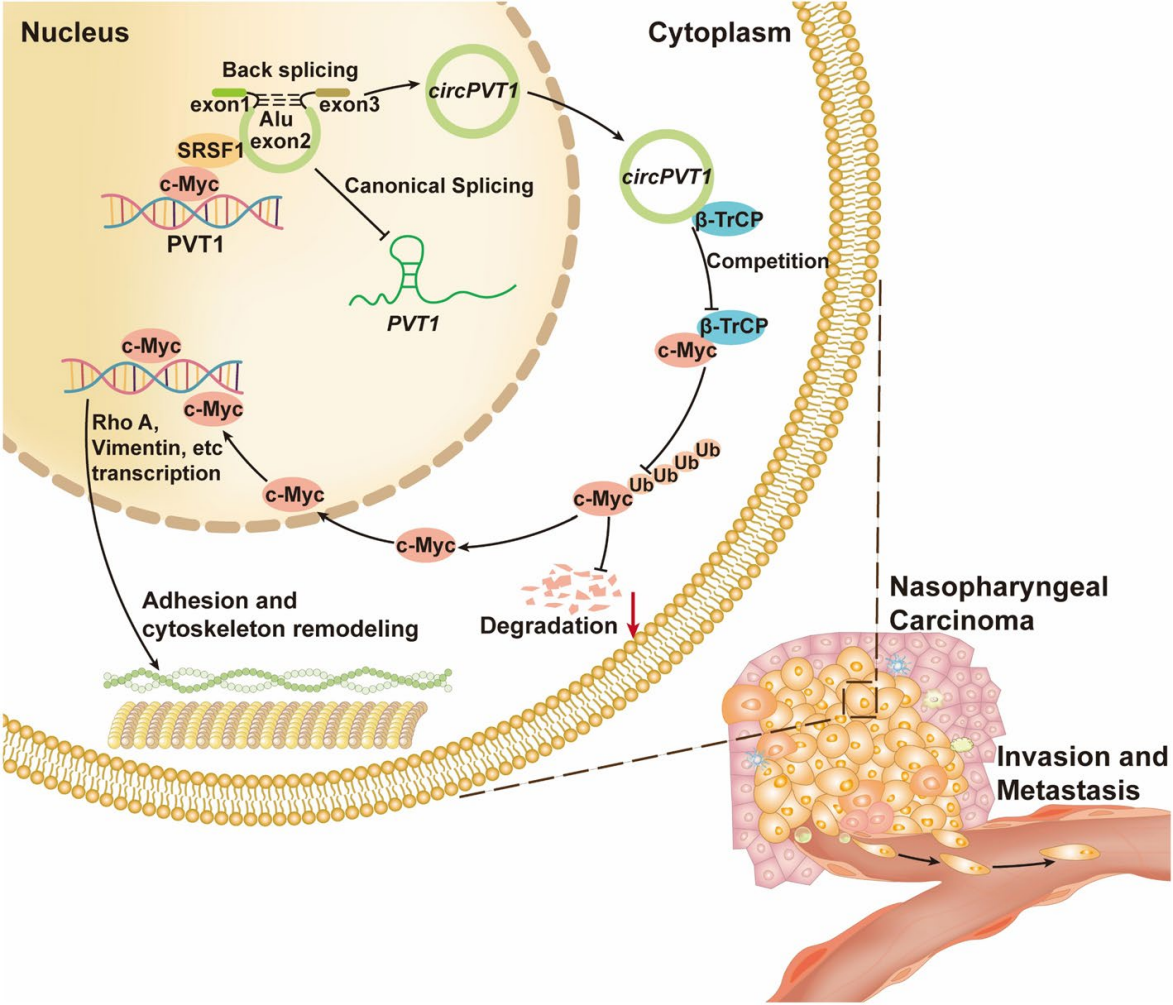
Schematic diagram of the molecular mechanism of *circPVT1* promoted NPC metastasis. Circular RNA *circPVT1* interacts with β-TrCP to prevent β-TrCP-induced c-Myc ubiquitination and degradation, which results in enhanced migration and invasion of NPC cells. Oncoprotein c-Myc promotes *circPVT1* transcription through binding to the promoter region of PVT1 gene, and recruits the splicing factor SRSF1 to facilitate *circPVT1* splicing, which forms a positive feedback loop that promotes the migration and invasion of NPC cells

In this study, we identified a circRNA *circPVT1* which was highly expressed in NPC cells and promoted NPC migration and invasion. Mechanistically, *circPVT1* interacted with β-TrCP to prevent β-TrCP-induced c-Myc ubiquitination and degradation, thus boosted the migration and invasion of NPC cells. Importantly, c-Myc not only promoted *PVT1* gene transcription through binding to the promoter region of *PVT1*, but also coordinated with the splicing factor SRSF1 to facilitate *circPVT1* biogenesis. These molecules form a positive feedback loop that enhances NPC migration and invasion (Fig. 8).

Amplification of chromosome 8q24, which *circPVT1* and lncRNA *PVT1* locate on, is frequently observed in a variety of cancers including NPC [20–24]. In this study, we focused on the circRNA *circPVT1* which was derived from exon 2 of the PVT1 gene and was highly expressed in NPC. The role of *circPVT1* in cancer was first reported in gastric cancer. Huang et al. found that *circPVT1* expression was elevated in gastric cancer and boosted the progression of gastric cancer [25]. Usually, *circPVT1* acts as a miRNA "sponge" to increase the expression of miRNA targeted mRNA at the post-transcriptional level to stimulate cancer progression [26–28]. In this work, we discovered that *circPVT1* could prevent β-TrCP-induced c-Myc ubiquitination and degradation through directly binding to β-TrCP protein, which ultimately promoted NPC cell migration and invasion.

As a key member ofthe F-box class of proteins, numerous studies have demonstrated that β-TrCP specifically recognizes and ubiquitinates IκB, β-catenin, and Emi1 proteins to regulate a variety of processes during tumor development [29, 30]. Here, we revealed that β-TrCP could bind and promote the degradation of c-Myc through ubiquitination. Interestingly, *circPVT1* competitively occupied the c-Myc-binding domain on β-TrCP to prevent β-TrCP-induced c-Myc ubiquitination and degradation, thus enhancing NPC cell migration and invasion. CircRNAs function as signaling pathway regulators in gene expression and other important cellular processes [31–36]. For example, circRNAs may act as scaffolds or decoys of RNA-binding proteins (RBPs) to form nuclear or cytoplasmic complexes [39–41]. Yang et al. found that *circFoxo3* in mice functions as a scaffold by binding to the cell-cycle proteins cyclin-dependent kinase 2 (CDK2) and cyclin-dependent kinase inhibitor 1 (or p21). The formation of a *circFoxo3*–p21–CDK2 ternary complex results in inhibition of CDK2 activity [37]. As a scaffold, *circFoxo3* also facilitates the degradation of mutant p53 and at the same time inhibits Foxo3 degradation by modulating double-minute 2 (MDM2)-mediated ubiquitination in murine cells [38]. Shan et al. found that *circBoule* RNAs interacts with conserved HSPs (heat shock proteins) and protects against stress-induced fertility decline [39]. Cai et al. found that *circPABPC1* negatively regulates adhesion and migration in hepatocellular carcinoma cells by directly binding to and down-regulating ITGB1 [40]. β-TrCP is a non-classical RNA binding protein, our results showed that *circPVT1* binds to β-TrCP and inhibits β-TrCP-mediated c-Myc protein degradation. Our study established an important theoretical basis for targeting circRNAs as therapeutic agents for c-Myc modulation, which is expected to fulfill the unmet clinical urgent needs.

Unexpectedly, we found that c-Myc could not only act as a transcription factor to drive gene transcription, but also bing in the splicing factor SRSF1 and facilitate transcriptional splicing of PVT1 into *circPVT1*, but not *PVT1*. Among a few studies on the generation of circRNA [41–47], Meng et al. found that transcription factor Twist1 promotes Cul2 transcription and up-regulates the expression of Cul2 circRNA through binding to Cul2 promoter [48]. Conn et al. found that the generation of circRNA was regulated by splicing factor Quaking (QKI) [49]. Wang et al. found that EIF4A3 could bind to MMP9 mRNA transcript to promote *circMMP9* cyclization and enhance *circMMP9* expression in glioblastoma [50]. Our work revealed a novel mechanism for upregulation of *circPVT1 *in NPC cells by coupling transcription with splicing event through synergetic corporation between c-Myc and SRSF1.

## Conclusions

Our study demonstrated that *circPVT1* was positively co-regulated by c-Myc and SRSF1 in NPC cells, and on the other hand *circPVT1* inhibited the ubiquitin-mediated degradation of c-Myc by binding to β-TrCP, which blocked the interaction between the ubiquitin E3 ligase β-TrCP and its target c-Myc. In turn, this led to cytoskeleton remodeling and cell adhesion modulation, and ultimately promoted NPC cell migration and invasion. Our work provided new insights into the mechanism of NPC progression and potential targets for the treatment of NPC patients.

## Supplementary Information

**Additional file 1:** **Supplemental Table 1.** Clinicopathological data of 60 NPC and 26 NPE tissues measured by qRT-PCR.

**Additional file 2:** **Supplemental Table 2.** Clinicopathological data of 159 paraffin-embedded NPC tissues and 29 non-neoplastic nasopharyngeal epithelialtissues for in situhybridization.

**Additional file 3**: **Supplemental Table 3.** Probes for fluorescence in situ hybridization and RNA pulldown, siRNAs, primers for qRT-PCR and vector construction.

**Additional file 4:** **Supplemental Table 4.** List of antibodies for immunohistochemistry, western blotting, immunofluorescence, and RNA pull-down experiments.

**Additional file 5**: **Supplemental Table 5.** The potential* circPVT1*-interacting proteins in CNE2 cells identified by LC-MS/MS spectrometry after pull-down with the biontin-labeled* circPVT1* probe.

**Additional file 6:** **Supplemental Table 6.** The potential β-TrCP-interacting proteins in CNE2 cells identified by LC-MS/MS spectrometry after pull-down with β-TrCP antibody.

**Additional file 7:** **Supplemental Table 7. **Proteomic analysis of *circPVT1*-regulated proteins in CNE2 cells after transfected with the *circPVT1*overexpression or empty vectors, followed by LC-MS/MS spectrometry.

**Additional file 8:** **Supplemental Table 8. **Clinicopathological data of 3 NPC tissues measured by RNA pull-down.

**Additional file 9:** **Supplemental Table 9. **Clinicopathologicaldata of 30 paraffin-embedded NPC tissues for immunohistochemistry (IHC) and in situ hybridization (ISH).

**Additional file 10:** **Figure S1.** A. Thedifferentially expressed circRNAs between the high metastasis NPC cell line S18 and the low metastasis NPC cell line S26 (GSE137543, the RPM value ≥ 2and Fold changes ≥ 1.5). Circular RNA *circPVT1* is highly expressed in NPC cell line S18.  B. The Venn diagram of data sets GSE137543 and PRJNA391554. Circular RNA *circPVT1* was identified for its high abundance in both sets of data.C.* circPVT1* is mainly localized in the cytoplasm as identified by nucleoplasmic separation experiments. U6 was used as a nuclear marker and GAPDH was used as a cytoplasmic marker.

**Additional file 11:** **Figure S2.*** circPVT1* promotes the migration and invasion of NPC in vitroand metastasis of NPCin vivo*. *A-B. The expression of *circPVT1* were measured in CNE2 and 5-8F cells after transfection with the *circPVT1* overexpression vector or* circPVT1* siRNA. *PVT1* expression was not affected in CNE2 and 5-8F cells after overexpresion or knockdown of *circPVT*. All experiments were repeated at least three times. Data were represented as mean ± SD. ***, *p* < 0.001; ns, not significant. C-D. Cell Counting Kit-8 and colony formation assays showed that *circPVT1* had no effect on the growth and proliferation of NPC cells. Data were represented as mean ± SD. ns, not significant. E. H&E staining of lung metastatic tumor foci and representative images of *circPVT1* expression as assessed by in situ hybridization (200×, scale bar, 50 μm). F. Images of footpad tumor formation in nude mice on day 21. Mice were injected with 1×10^6^ CNE2 cells after overexpression or knockdown of *circPVT1*.G. Representative images of immunohistochemical staining for pan-cytokeratin in lymphatic tissues of mice. Scale bar, 50 μm.

**Additional file 12: Figure S3.** *circPVT1 *promotes the migration and invasion of NPC cells through binding to β-TrCP. A. The secondary ion flow diagram of β-TrCP identified by mass spectrometry according to the peptide sequence (DFITALPAR) of β -TrCP.B. Schematic diagram of binding sites betweent* circPVT1* and β-TrCP using the catRAPID website.The 230-280 nt of *circPVT1* was predicted to bind to β-TrCP protein with high interaction score.C. The secondary structure of *circPVT1* changed when 230-280 nt of *circPVT1* was deleted (△circPVT1).D. Wound healing assays showed that the wild type *circPVT1* but not the mutant (△circPVT1) reduced the migration abilities of CNE2 and 5-8F cells after transfected with the full-length *circPVT1* or the mutant (230-280 nt deleted, △circPVT1). Data were represented as mean ± SD. ***, *p* < 0.001, ns, not significant. E. Transwell invasion assays showed that the wild type *circPVT1* but not mutant of *circPVT1* (△circPVT1) reduced the migration abilities of CNE2 and 5-8F cells wereafter transfected with the full-length *circPVT1* or the mutant (230-280 nt deleted, △circPVT1). Data were represented as mean ± SD. ***, *p* < 0.001, ns, not significant.

**Additional file 13:** **Figure S4. **The WD40 repeat domain of β-TrCP interacts with *circPVT1.* A. The functional domain of the of β-TrCP proteins are illustrated (top), including the F-box domain and the WD40 domain. The expressing plasmids for full-length β-TrCP or truncated fragments (F-box and WD40) were constructed and confirmed using anti-Flag antibody. B. Wound healing assay showed that overexpression of β-TrCP inhibited NPC cells migration. Data were represented as mean ± SD. ***, *p* < 0.001, ns, not significant. C. Transwell invasion assay showed that overexpression of β-TrCP inhibited NPC cells invasion. Data were represented as mean ± SD. ***, *p* < 0.001, ns, not significant.

**Additional file 14:** **Figure S5.** c-Myc is ubiquitinated substrate of β-TrCP. A. The expression of β-TrCP was examined in CNE2 and 5-8F cells after overexpression or knockdown of *circPVT1* using western blotting.B. The β-TrCP binding proteins were identified in CNE2 cells by immunoprecipitation followed by LC-MS/MS.C. The interaction of c-Myc and β-TrCP was predicted by Molecular Docking (http://hdock.phys.hust.edu.cn/). The blue part is the WD40 repeat domain of β-TrCP.D. The expression of c-Myc was examined in CNE2 and 5-8F cells after overexpression of β-TrCP using western blotting.E. The ubiquitination level of c-Myc protein was determined in CNE2 and 5-8F cells after overexpression of β-TrCP and immunoprecipitation with anti-c-Myc antibody, followed by western blotting with an anti-ubiquitin antibody.

**Additional file 15:** **Figure S6.** *circPVT1* promotes the migration and invasion of NPC cells by regulating cell adhesion andcytoskeleton remodeling. The 231 differentially expressed proteins regulated by c-Myc from LC-MS/MS data using the Ingenuity Pathway Analysis (IPA) software.

**Additional file 16:** **Figure S7. **c-Myc promotes *circPVT1* generation by recruiting SRSF1 to couple transcription to splicing. A. The luciferase reporter gene activity of the PVT1 promoter was analyzed in NPC cells after overexpression of c-Myc. Data were represented as mean ± SD. ***, *p* < 0.001. B. The binding of transcription factor c-Myc to the PVT1 promoter detected by ChIP assay. Three sites on the PVT1 promoter were identified as potential c-Myc binding sites (site 1, site 2 and site 3) using the Jasper and PROMO software. Data were represented as mean ± SD. ***, *p* < 0.001, ns, not significant. C. The luciferase reporter gene activity of the SRSF1 promoter was analyzed in NPC cells after overexpression of c-Myc. Data were represented as mean ± SD. ***, *p* < 0.001. D. The binding of transcription factor c-Myc to the PVT1 promoter detected by ChIP assay. Two sites on the PVT1 promoter were selected as the c-Myc binding sites (site 1 and site 2) using the Jasper and PROMO software. Data were represented as mean ± SD. ***, *p* < 0.001, ns, not significant. E. Binding of *circPVT1* to β-TrCP protein was analyzed in NPC tissues after pulling-down with biotin-labeled *circPVT1* probe. The biotin-labeled scrambled sequences was used as a control.F. Representative images showing the correlation between *circPVT1* and the expression of c-Myc and SRSF1 in NPC tissues (top). Quantification of the correlation between *circPVT1* and the expression of c-Myc and SRSF1 in NPC tissues (bottom). Magnification: 200×, Scale bar = 50 μm, 400×, Scale bar = 20 μm.

## Data Availability

All data that support the findings of this study are available from the corresponding author upon request.

## Abbreviations

NPC: Nasopharyngeal carcinoma
circRNA: Circular RNAs
PVT1: Plasmacytoma variant translocation 1
β-TrCP: Beta-transducin repeat containing E3 ubiquitin protein ligase
LC–MS/MS: Liquid Chromatography-tandem Mass Spectrometry
IPA: Ingenuity Pathway Analysis
RhoA: Ras homolog family member A
RhoC: Ras homolog family member C
GAPDH: Glyceraldehyde-3-phosphate dehydrogenase
SRSF1: Serine and arginine rich splicing factor 1
