# Supplementary figures and images for: Circular RNA circPVT1 promotes nasopharyngeal carcinoma metastasis via the β-TrCP/c-Myc/SRSF1 positive feedback loop
Source: Mol Cancer. 2022 Oct 5;21:192. doi: 10.1186/s12943-022-01659-w (PMC9533486; doi:10.1186/s12943-022-01659-w)

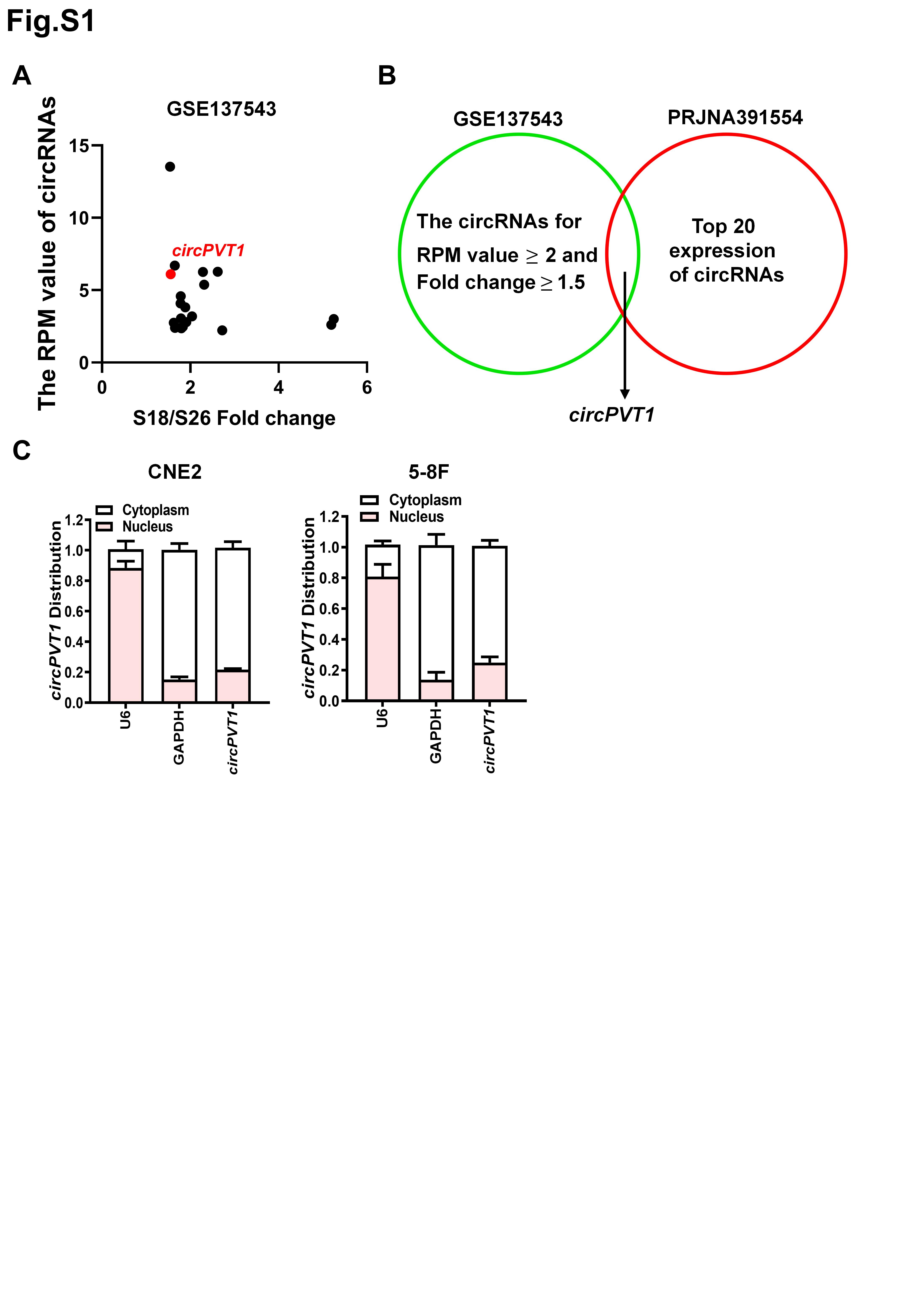

Supplement: Supplementary file 10 — Additional file 10: Figure S1. A. Thedifferentially expressed circRNAs between the high metastasis NPC cell line S18 and the low metastasis NPC cell line S26 (GSE137543, the RPM value ≥ 2and Fold changes ≥ 1.5). Circular RNA circPVT1 is highly expressed in NPC cell line S18. B. The Venn diagram of data sets GSE137543 and PRJNA391554. Circular RNA circPVT1 was identified for its high abundance in both sets of data.C. circPVT1 is mainly localized in the cytoplasm as identified by nucleoplasmic separation experiments. U6 was used as a nuclear marker and GAPDH was used as a cytoplasmic marker. [file 12943_2022_1659_MOESM10_ESM.jpg]

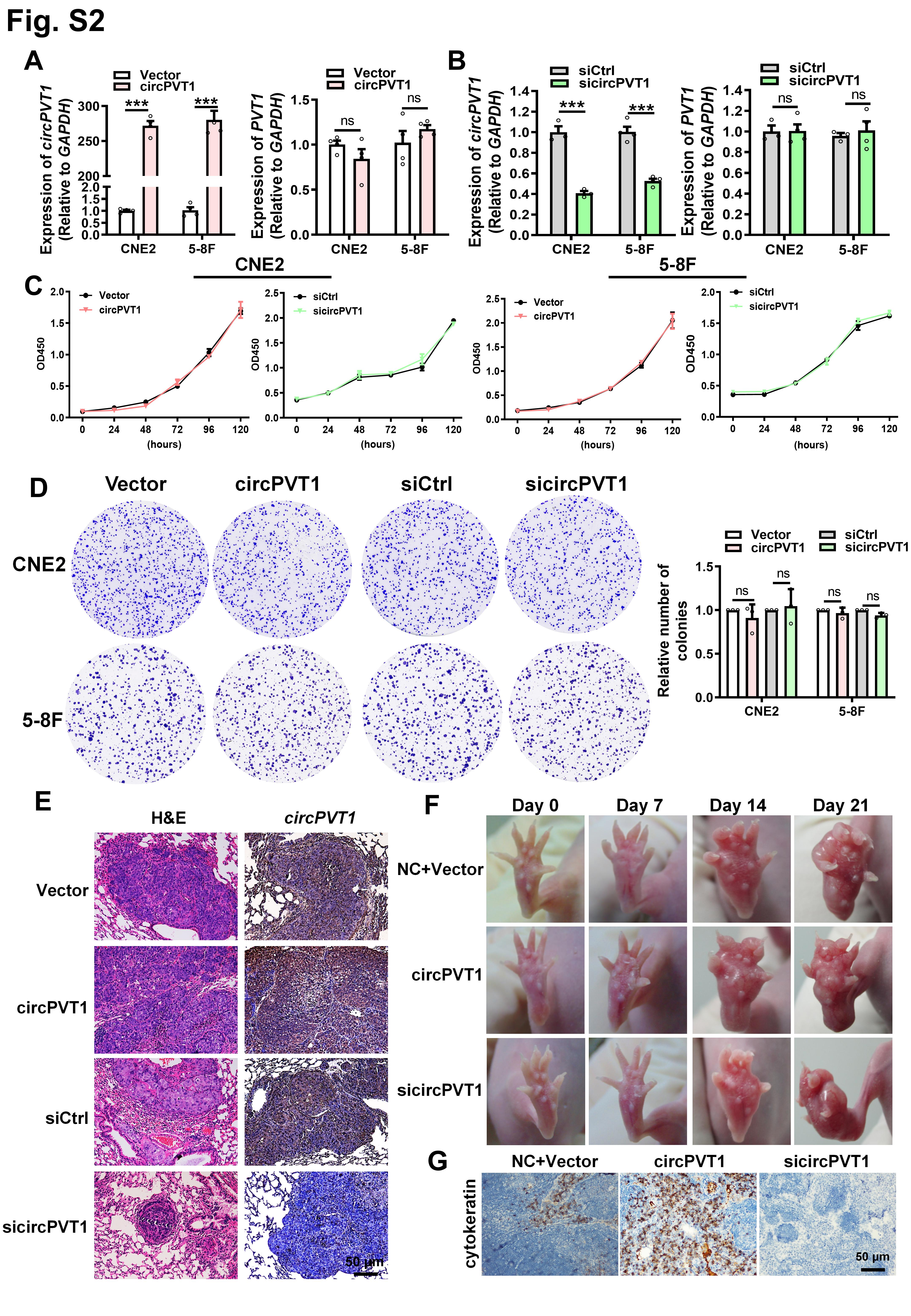

Supplement: Supplementary file 11 — Additional file 11: Figure S2. circPVT1 promotes the migration and invasion of NPC in vitroand metastasis of NPCin vivo. A-B. The expression of circPVT1 were measured in CNE2 and 5-8F cells after transfection with the circPVT1 overexpression vector or circPVT1 siRNA. PVT1 expression was not affected in CNE2 and 5-8F cells after overexpresion or knockdown of circPVT. All experiments were repeated at least three times. Data were represented as mean ± SD. ***, p < 0.001; ns, not significant. C-D. Cell Counting Kit-8 and colony formation assays showed that circPVT1 had no effect on the growth and proliferation of NPC cells. Data were represented as mean ± SD. ns, not significant. E. H&E staining of lung metastatic tumor foci and representative images of circPVT1 expression as assessed by in situ hybridization (200×, scale bar, 50 μm). F. Images of footpad tumor formation in nude mice on day 21. Mice were injected with 1×10^6^ CNE2 cells after overexpression or knockdown of circPVT1.G. Representative images of immunohistochemical staining for pan-cytokeratin in lymphatic tissues of mice. Scale bar, 50 μm. [file 12943_2022_1659_MOESM11_ESM.jpg]

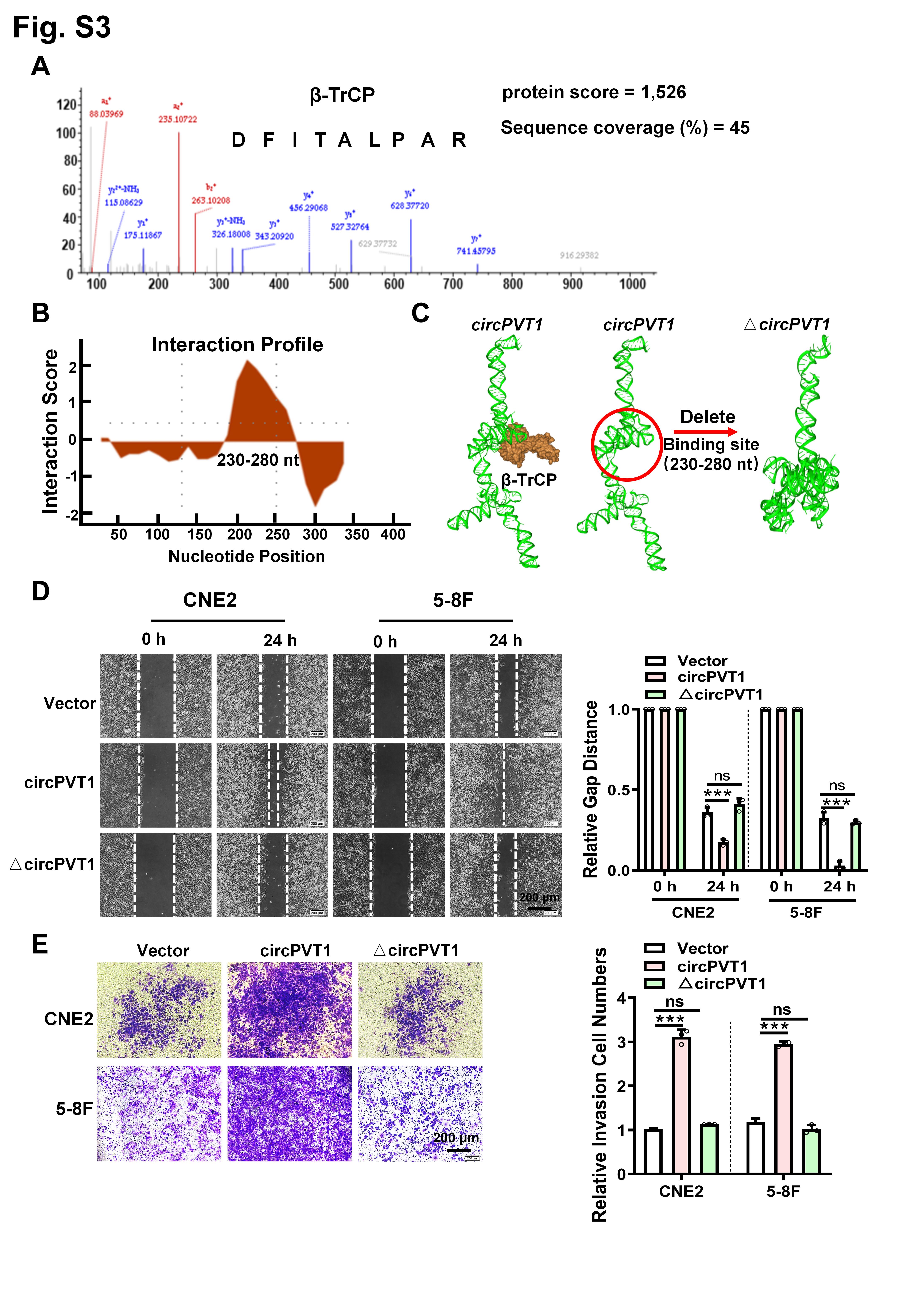

Supplement: Supplementary file 12 — Additional file 12: Figure S3. circPVT1 promotes the migration and invasion of NPC cells through binding to β-TrCP. A. The secondary ion flow diagram of β-TrCP identified by mass spectrometry according to the peptide sequence (DFITALPAR) of β -TrCP.B. Schematic diagram of binding sites betweent circPVT1 and β-TrCP using the catRAPID website.The 230-280 nt of circPVT1 was predicted to bind to β-TrCP protein with high interaction score.C. The secondary structure of circPVT1 changed when 230-280 nt of circPVT1 was deleted (△circPVT1).D. Wound healing assays showed that the wild type circPVT1 but not the mutant (△circPVT1) reduced the migration abilities of CNE2 and 5-8F cells after transfected with the full-length circPVT1 or the mutant (230-280 nt deleted, △circPVT1). Data were represented as mean ± SD. ***, p < 0.001, ns, not significant. E. Transwell invasion assays showed that the wild type circPVT1 but not mutant of circPVT1 (△circPVT1) reduced the migration abilities of CNE2 and 5-8F cells wereafter transfected with the full-length circPVT1 or the mutant (230-280 nt deleted, △circPVT1). Data were represented as mean ± SD. ***, p < 0.001, ns, not significant. [file 12943_2022_1659_MOESM12_ESM.jpg]

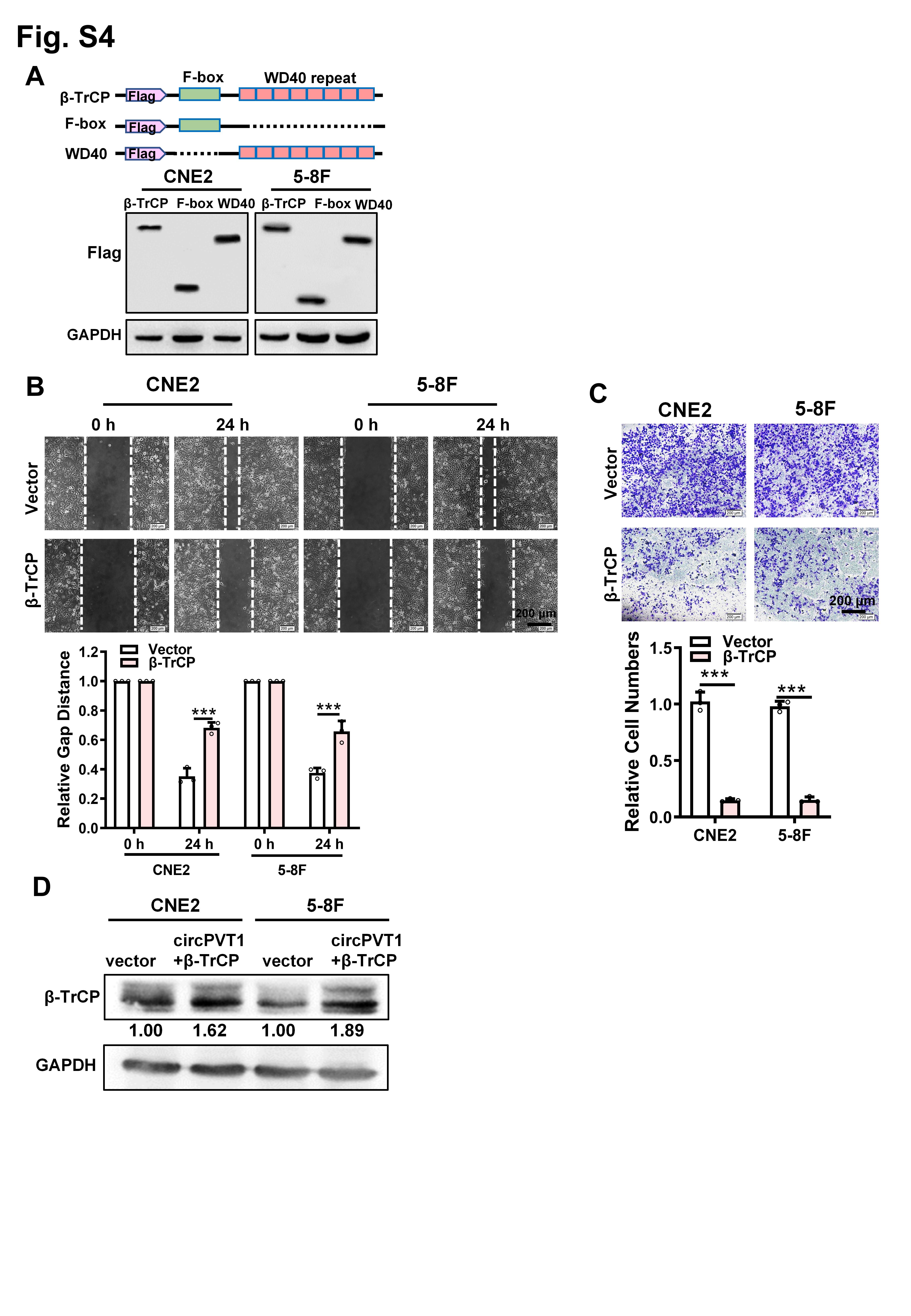

Supplement: Supplementary file 13 — Additional file 13: Figure S4. The WD40 repeat domain of β-TrCP interacts with circPVT1. A. The functional domain of the of β-TrCP proteins are illustrated (top), including the F-box domain and the WD40 domain. The expressing plasmids for full-length β-TrCP or truncated fragments (F-box and WD40) were constructed and confirmed using anti-Flag antibody. B. Wound healing assay showed that overexpression of β-TrCP inhibited NPC cells migration. Data were represented as mean ± SD. ***, p < 0.001, ns, not significant. C. Transwell invasion assay showed that overexpression of β-TrCP inhibited NPC cells invasion. Data were represented as mean ± SD. ***, p < 0.001, ns, not significant. [file 12943_2022_1659_MOESM13_ESM.jpg]

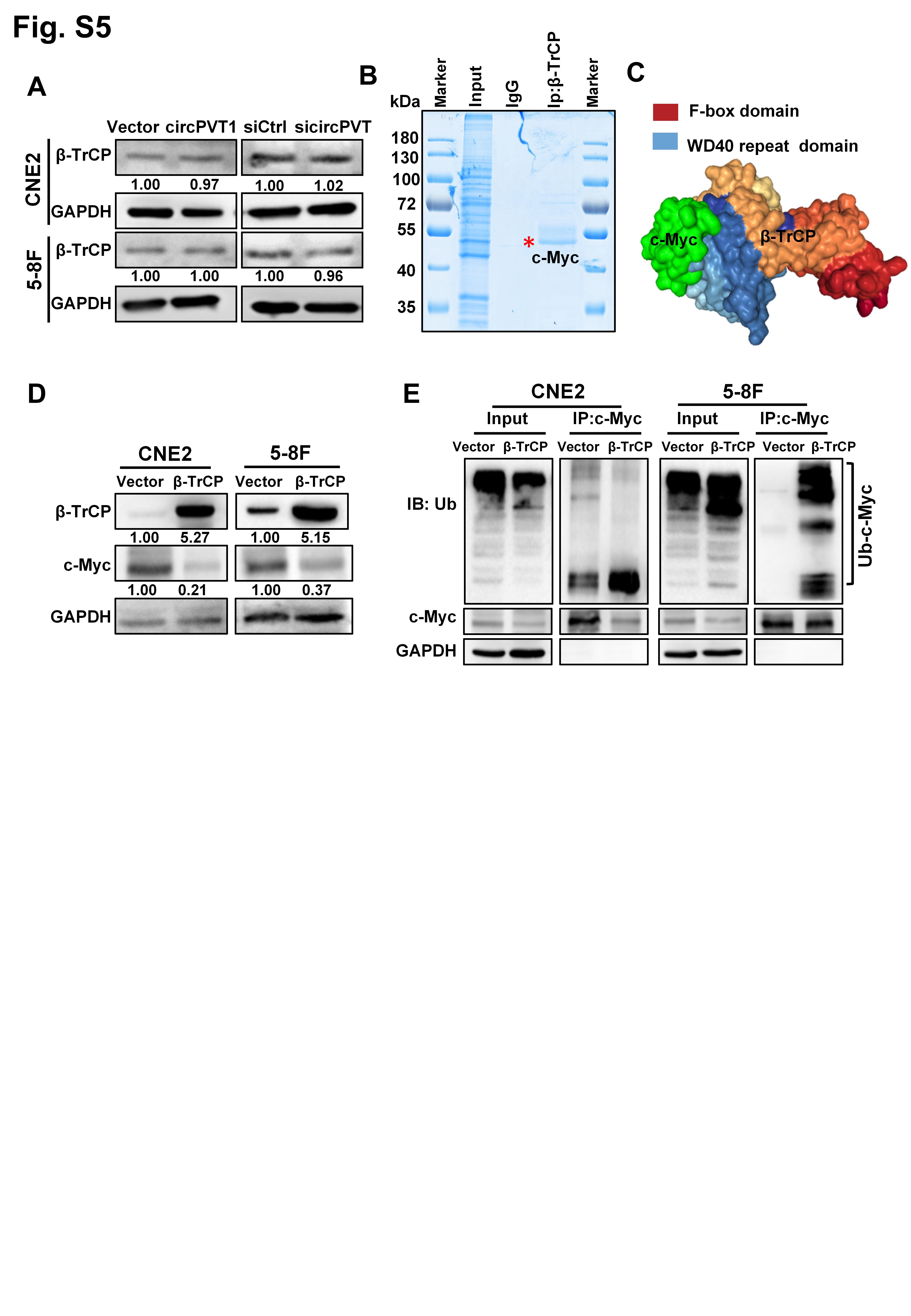

Supplement: Supplementary file 14 — Additional file 14: Figure S5. c-Myc is ubiquitinated substrate of β-TrCP. A. The expression of β-TrCP was examined in CNE2 and 5-8F cells after overexpression or knockdown of circPVT1 using western blotting.B. The β-TrCP binding proteins were identified in CNE2 cells by immunoprecipitation followed by LC-MS/MS.C. The interaction of c-Myc and β-TrCP was predicted by Molecular Docking (http://hdock.phys.hust.edu.cn/). The blue part is the WD40 repeat domain of β-TrCP.D. The expression of c-Myc was examined in CNE2 and 5-8F cells after overexpression of β-TrCP using western blotting.E. The ubiquitination level of c-Myc protein was determined in CNE2 and 5-8F cells after overexpression of β-TrCP and immunoprecipitation with anti-c-Myc antibody, followed by western blotting with an anti-ubiquitin antibody. [file 12943_2022_1659_MOESM14_ESM.jpg]

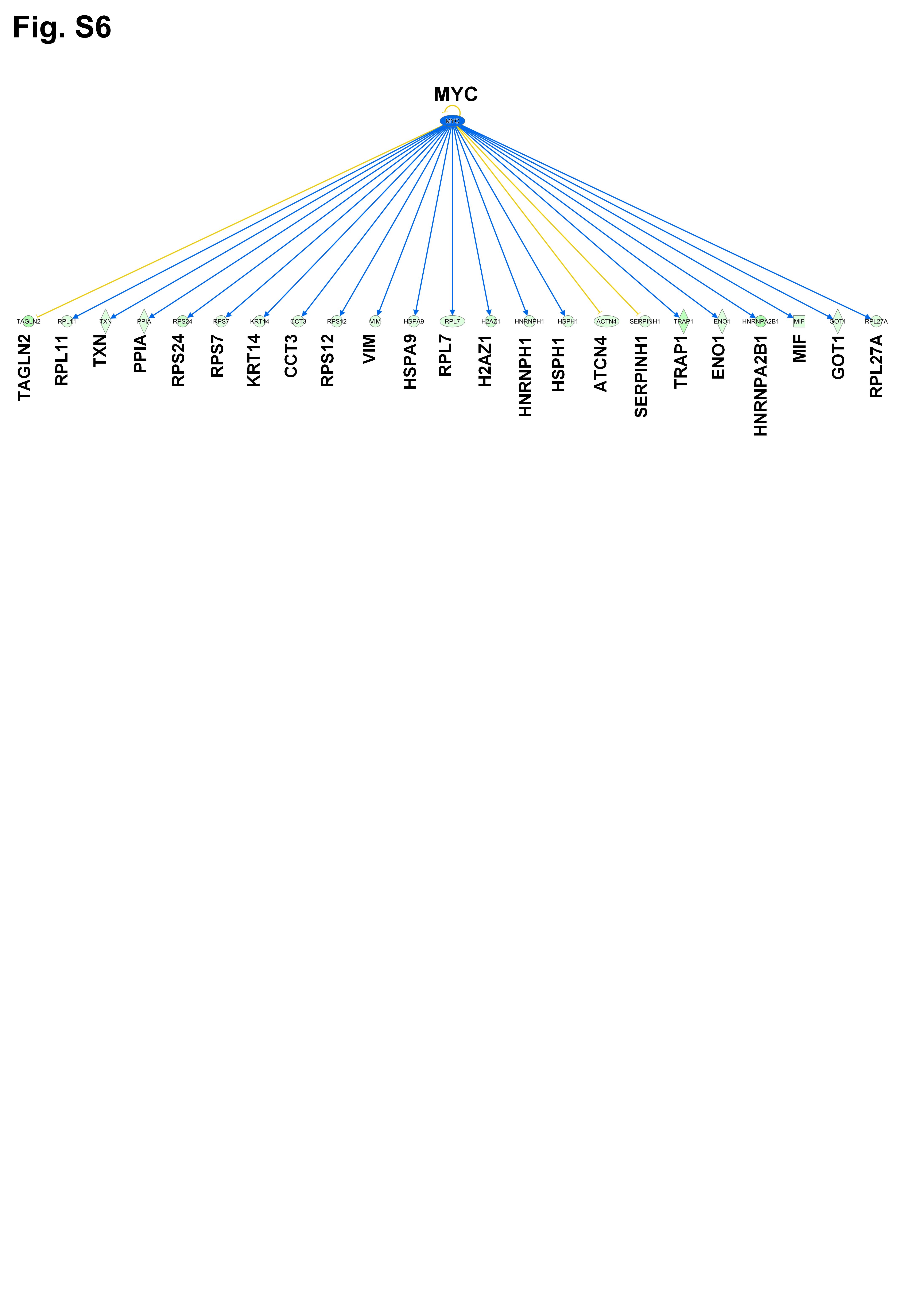

Supplement: Supplementary file 15 — Additional file 15: Figure S6. circPVT1 promotes the migration and invasion of NPC cells by regulating cell adhesion andcytoskeleton remodeling. The 231 differentially expressed proteins regulated by c-Myc from LC-MS/MS data using the Ingenuity Pathway Analysis (IPA) software. [file 12943_2022_1659_MOESM15_ESM.jpg]

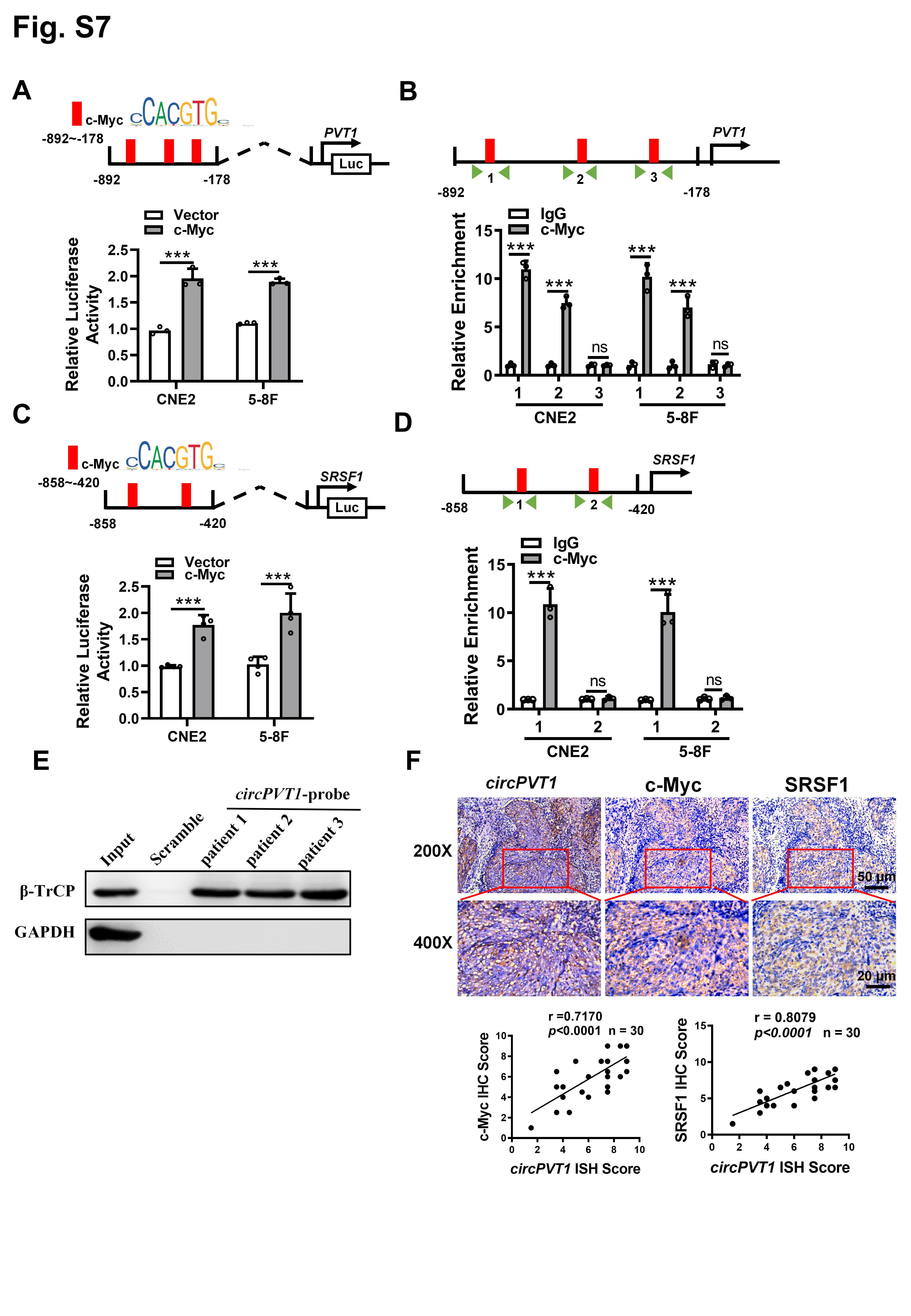

Supplement: Supplementary file 16 — Additional file 16: Figure S7. c-Myc promotes circPVT1 generation by recruiting SRSF1 to couple transcription to splicing. A. The luciferase reporter gene activity of the PVT1 promoter was analyzed in NPC cells after overexpression of c-Myc. Data were represented as mean ± SD. ***, p < 0.001. B. The binding of transcription factor c-Myc to the PVT1 promoter detected by ChIP assay. Three sites on the PVT1 promoter were identified as potential c-Myc binding sites (site 1, site 2 and site 3) using the Jasper and PROMO software. Data were represented as mean ± SD. ***, p < 0.001, ns, not significant. C. The luciferase reporter gene activity of the SRSF1 promoter was analyzed in NPC cells after overexpression of c-Myc. Data were represented as mean ± SD. ***, p < 0.001. D. The binding of transcription factor c-Myc to the PVT1 promoter detected by ChIP assay. Two sites on the PVT1 promoter were selected as the c-Myc binding sites (site 1 and site 2) using the Jasper and PROMO software. Data were represented as mean ± SD. ***, p < 0.001, ns, not significant. E. Binding of circPVT1 to β-TrCP protein was analyzed in NPC tissues after pulling-down with biotin-labeled circPVT1 probe. The biotin-labeled scrambled sequences was used as a control.F. Representative images showing the correlation between circPVT1 and the expression of c-Myc and SRSF1 in NPC tissues (top). Quantification of the correlation between circPVT1 and the expression of c-Myc and SRSF1 in NPC tissues (bottom). Magnification: 200×, Scale bar = 50 μm, 400×, Scale bar = 20 μm. [file 12943_2022_1659_MOESM16_ESM.jpg]
